# Sensory focused exercise improves anxiety in Parkinson’s disease: A randomized controlled trial

**DOI:** 10.1371/journal.pone.0230803

**Published:** 2020-04-16

**Authors:** Eric N. Beck, Mary T. Y. Wang, Brittany N. Intzandt, Quincy J. Almeida, Kaylena A. Ehgoetz Martens

**Affiliations:** 1 School of Medicine, Trinity College Dublin, The University of Dublin, Dublin, Ireland; 2 Department of Kinesiology and Physical Education, Movement Disorders Research & Rehabilitation Centre, Wilfrid Laurier University, Waterloo, Ontario, Canada; 3 Cleveland Clinic, Cleveland, Ohio, United States of America; 4 Cognitive Health and Aging Research Lab, Concordia University, Montreal, Quebec, Canada; 5 Department of Kinesiology, University of Waterloo, Waterloo, Ontario, Canada; Cardiff University, UNITED KINGDOM

## Abstract

Anxiety has been implicated as one of the greatest influences on quality of life in Parkinson’s disease (PD). The etiology of anxiety is unclear, although previous work suggests that anxiety may be linked to sensory deficits that cause uncertainty in movement. Thus, the current study examined whether focusing attention on sensory feedback during goal-based exercise has the potential to provide benefits to anxiety in PD. Thirty-five participants with PD were randomized to either a Sensory Attention Focused Exercise (SAFEx) (i.e. internal focus of attention, n = 18) or Sham Exercise control (i.e. external focus of attention, n = 17) and completed 33 one-hour attention-based exercise sessions over 11-weeks. Before and after the program (pre and post), participants completed the Parkinson Anxiety Scale (PAS) questionnaire. The PAS includes three anxiety sections: persistent, episodic, and avoidance. Changes in the total PAS score and within each section of the PAS were subjected to two-factor mixed repeated measures ANCOVA. Significant group by time interactions demonstrated that from pre to post, total PAS scores (p = 0.007) and episodic anxiety scores (p = 0.010) significantly decreased in the SAFEx group only (ΔTotal PAS = -5.2, F_(1,27)_ = 5.41, p = 0.028, η_p_^2^ = 0.17; ΔEpisodic Score = -1.8, F_(1,27)_ = 6.89, p = 0.014, η_p_^2^ = 0.20). In conclusion, focusing attention on sensory feedback while completing goal-based exercises may provide significant benefits to improving anxiety in PD. As such, sensory attention focused exercise may be a critical adjunct therapy for improving anxiety, and ultimately quality of life in people with PD.

## Introduction

Anxiety affects up to 6% of patients with Parkinson’s disease (PD) [1,2]. As such, it is not surprising that patients and caregivers rank anxiety as the most debilitating symptom requiring improved management and therapy, second only to falls and balance impairments [3]. Although the etiology of anxiety in PD remains unclear [4], anxiety is a non-motor symptom that affects PD patients early on in the disease course, often predating motor symptoms [5], and has been linked with sensory symptoms and sensory deficits [6–8].

Sensory deficits (e.g. proprioceptive impairments) are a well-known non-motor symptom that accompanies PD, and disturbs balance and movement control [9–12]. As sensorimotor integration becomes increasingly impaired, one could postulate that greater uncertainty in sensory feedback might arise. Over time, this may result in less confidence and reliability in one’s movement, and ultimately manifest greater levels of anxiety. Evidence from healthy individuals and PD patients suggests that anxiety has persistent effects on attention and disrupts working memory [13]. Given that individuals with PD progressively lose their ability to perform motor tasks without conscious control (in part due to impaired processing of sensory information), PD patients develop an increased reliance on attention to guide movement relative to healthy individuals [11,14,15–22,23]. In this sense, anxiety may have an even greater detrimental effect on one’s ability to compensate for sensory deficits to control movement.

Recent evidence has demonstrated a relationship between anxiety and movement control. For instance, individuals with PD who report high trait anxiety have greater balance and gait impairments [8,24,25], as well as increased susceptibility to dual task interference compared to non-anxious individuals with PD [26]. Another study showed that when sensory feedback was provided in virtual reality, which simulated walking across a plank, anxiety had a reduced effect on walking compared to when sensory feedback was not provided [8]. These results align with the notion that directing attention to reduce sensory uncertainty may reduce the influence of anxiety on movement. Overall, the interaction between sensory feedback, attention and anxiety may be crucial contributors to movement impairments in PD, and thus targeting attentional focused exercise to reduce sensory uncertainty may be a useful rehabilitative therapy for improving anxiety in PD.

To date, multiple studies have aimed to improve anxiety in PD with pharmacological interventions, however randomized controlled trials have demonstrated that selective serotonin reuptake inhibitors and tricyclic antidepressants have not successfully improved anxiety beyond a placebo, and have been often coupled with various negative side effects [27–31]. Thus, other potential interventions and adjunct therapies for anxiety in PD also require investigation, and exercise may be a promising possibility.

Meta-analyses have demonstrated that physical activity can provide significant benefits to anxiety in various clinical populations [32–36]. However, to date, knowledge regarding the influence of exercise on anxiety in individuals with PD is limited. Dashtipour and colleagues (2015) found that a goal-based exercise program and a general exercise program (treadmill and seated upper extremity exercises) significantly improved anxiety as a secondary outcome in a small sample of PD patients [37]. Furthermore, Clarke and colleagues (2009) found subtle improvements in anxiety after exercise in a pilot study, but did not subject their findings to statistical analysis [38]. Thus, a randomized controlled trial investigating the effect of exercise on anxiety in PD is desperately needed. To specifically improve upon anxiety, one possibility may be goal-based exercise rehabilitation that aims to pull attention towards impaired movements while they are performed.

Throughout movement performance, individuals can direct their focus of attention ‘internally’ towards either sensory information regarding limb position (i.e. proprioception), or ‘externally’ towards the effect of an action on the environment [39]. Previous work has demonstrated that focusing attention on sensory feedback throughout a goal-based exercise program (Parkinson’s disease Sensory Attention Focused Exercise, PD-SAFEx^™^) provided benefits to balance and gait, potentially by improving upon one’s ability to consciously process faulty sensory feedback [40–42]. If anxiety is linked to sensory processing limitations, then focusing attention on sensory feedback during a goal-based exercise program may provide benefits to anxiety. Therefore, the present randomized controlled trial aimed to investigate whether sensory attention focused exercise might improve upon validated measures of anxiety in PD.

## Materials and methods

### Participants

Participants were randomly recruited from the Movement Disorders Research and Rehabilitation Centre (MDRC) exercise database at Wilfrid Laurier University, Waterloo, Canada. Individuals diagnosed with idiopathic PD by a neurologist, of either gender were included in the present study, providing they could understand simple verbal English instructions, and were able to stand for 5 minutes and walk 10-metres without assistance. Participants were excluded if diagnosed with dementia, diabetes, peripheral neuropathy, or a neurological disease other than PD. Individuals participating in an exercise routine prior to starting the present exercise program were not excluded. In accordance with the Declaration of Helsinki, written informed consent was obtained from all participants prior to any participation in this study. Ethical approval was obtained from the Wilfrid Laurier University Research Ethics Board. Prior to starting the rehabilitation interventions, all participants were required to obtain a completed Physical Activity Readiness Medical Examination (ParMed X) form signed by a medical doctor. The present study was registered with the U.S. National Institutes of Health (ClinicalTrials.gov Identifier: NCT02476240).

### Study design and exercise intervention

The present study utilized a single blind (assessments before and after the intervention were conducted by evaluators blinded to group allocation), parallel group, randomized controlled trial design. All individuals with PD interested in participating in the study, and who fit the inclusion criteria, were asked to visit the MDRC one week prior to the scheduled start-date of the exercise program for assessment with primary and secondary outcome measures (pre assessment). After pre assessment was concluded, participants were randomized (via computerized randomization conducted by the MDRC laboratory coordinator) into one of two groups: a sensory attention focused exercise group (SAFEx) or a sham exercise control (Sham Exercise) group. Participants were informed of group allocation via telephone call made by the MDRC laboratory coordinator. The only individuals blinded to the allocation of the participants were the investigator conducting pre and post testing, and the movement disorders specialist whom conducted all UPDRS-III assessments. These individuals were not present for the weekly exercise program and not informed of group allocation at any point.

Participants in the SAFEx group were continually instructed to focus their attention towards the sensory feedback of their limbs in physical space during the required movements for the exercise program. For example, during a knee raise, participants were consistently given instructions to “focus on moving their knee up in a slow, controlled manner”. Conversely, the Sham Exercise group completed all exercises while directing their attention toward the movement of coloured labels attached to their limbs (red labels over the dorsum of each hand, blue labels over the dorsum of each foot, green labels over the superior patella, and yellow labels over the medial epicondyle of the humerus). Directing attention towards labels attached to one’s body has been demonstrated to guide movement through the external environment and effectively take away the focus from ‘internal’ sensory feedback, resulting in a change in movement performance [43]. Participants were consistently and continually given instructions to focus on the movement of the coloured labels; for example, during a knee raise, participants were instructed to “focus on pushing the green label up through the air in a slow, controlled manner.”

To control for learning effects and bias, and to demonstrate natural disease progression without changes to daily routine (such as introducing an exercise program), the study design included a non-exercise control group of individuals who completed pre and post assessment evaluations, but expressed that they were unable to commit to the program. Thus, participants were not randomized to the non-exercise control group. This was the case for 3 reasons. Firstly, we aimed to provide exercise to any members of the community with PD whom wished to be involved. Secondly, withholding exercise from people enrolled into the MDRC exercise database of participants with PD was deemed unethical by the Wilfrid Laurier University ethics board. Thirdly, attrition rates/dropouts of individuals randomly assigned to a control group often leave a small number. Since the purpose of the non-exercise control group was only to serve as an important reference point and they were not randomized, this group was not included in the statistical analysis.

**Fig 1:**
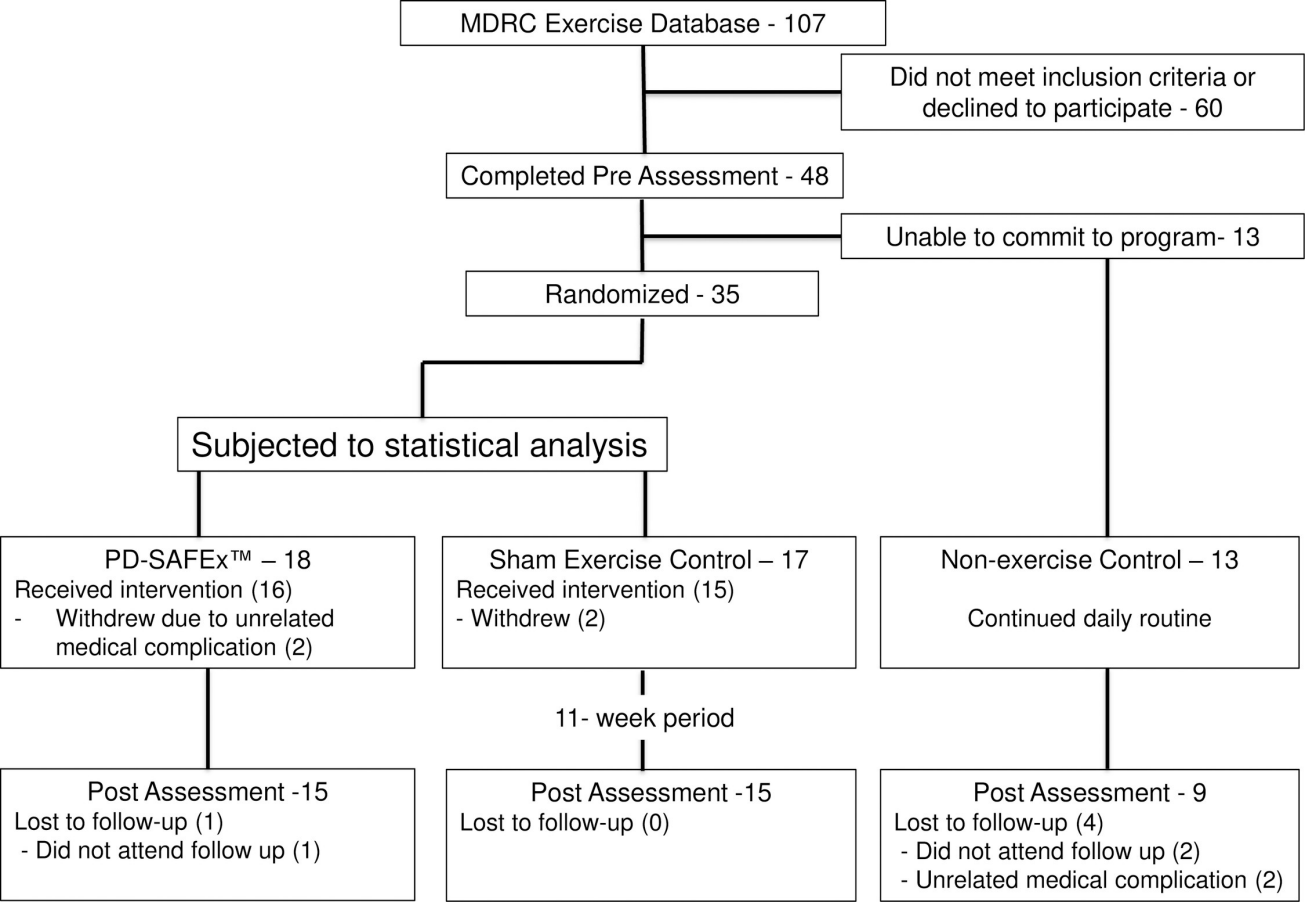
Profile flow chart. MDRC = Movement Disorders Research and Rehabilitation Centre; PD-SAFEx = Parkinson’s Disease Sensory Attention Focused Exercise.

The Parkinson’s disease Sensory Attention Focused Exercise (PD-SAFEx^TM^) program was utilized for the present randomized controlled trial [40]. The program began one week after pre assessment, and lasted 11-weeks. PD-SAFEx^™^ is a goal-based exercise program that includes stretching, coordination, balance, and gait training. Participants completed three one-hour sessions per week for the 11-week program duration. Training sessions were completed in a group setting, and the difficulty of exercises progressively increased every consecutive week to challenge participants’ coordination and balance as their proficiency progressed. The program was completed by both exercise groups identically in terms of the type of movements, number of sets and repetitions. The groups differed only in the attentional instruction they were given (to focus either on sensory feedback from their limbs throughout each movement [SAFEx], or away from sensory feedback towards moving coloured labels [Sham Exercise]). Participants began exercise sessions approximately one hour after taking their dopaminergic medication. A graduate student with a background in kinesiology instructed and supervised all exercise classes. A number of volunteer undergraduate kinesiology students aided participants for safety and ensured proper form during the exercises. Post assessments took place the week immediately following the last week of the exercise program. Despite instructions to refrain from making changes to medications throughout the study, the levodopa equivalent dose (LED) was calculated at pre and post assessment to confirm whether any changes in levodopa treatment had occurred. Please see the profile flow chart in Fig 1 for a full breakdown of participant recruitment, randomization, assessment time points, and withdrawals from the study.

### Outcome measures

#### Primary outcome measures

To assess anxiety in patients before and after the intervention timeframe, the Parkinson Anxiety Scale (PAS) [44] and the State and Trait Anxiety Inventory (STAI) [45] were utilized. The PAS is a new tool developed specifically to assess anxiety in Parkinson’s patients. Regarding reliability, Cronbach’s alpha demonstrated a total PAS observer rating of 0.87 and self-rating of 0.89. Inter-rater reliability of the total PAS was calculated 0.92 and test-retest reliability 0.89. Regarding validity, total PAS area under the curve (AUC), sensitivity, and specificity for observer rated PAS were 85.9, 0.71, and 0.91, respectively. Self-rated total PAS AUC, sensitivity, and specificity was 85.1, 0.81, and 0.74, respectively. Subsections of the PAS demonstrated similar reliability and validity values [44]. Thus to date, the PAS has been labelled as the gold standard to assess anxiety in PD. However, one limitation of this scale is that its sensitivity to change has not been formally evaluated longitudinally. This was one reason the STAI was also included, since it has been extensively validated in healthy populations, has been suggested for use in PD by the Movement Disorder Society, and has been proven to be sensitive to change in numerous studies [46–48].

#### Secondary outcome measures

General cognitive capability (Montreal Cognitive Assessment; MOCA) [49], inhibition (Stroop task) [50], set-shifting (Trail-Making task) [51], and phonemic fluency (Verbal fluency task) [52] were measured to assess cognitive functioning at pre and post assessments. To achieve a thorough assessment of participants’ symptom severity, and gain an indication of basal ganglia functioning not masked by exogenous dopamine replacement, the Unified Parkinson’s Disease Rating Scale Motor Section (UPDRS Sub-section III) was utilized while participants were both OFF (>12 hour withdrawal from dopaminergic medication) and ON (1 hour after taking their normal dopaminergic medication) their dopaminergic medication. Participants’ perceived quality of life was quantified with the 39-item Parkinson’s disease Questionnaire (PDQ-39) [53]. Lastly, in order to quantify physical activity levels 4 weeks prior to the initiation of the interventions (to control for activity levels prior to commencement in the exercise program), the validated Community Health Activities Model Program for Seniors questionnaire (CHAMPS) was utilized [54]. This measure, an estimated caloric expenditure calculated by multiplying the estimated duration of each activity by the estimated MET value, allowed determination of all physical activity levels (specified by the measure) and moderate intensity (≥3 METs) physical activity levels prior to and during the last 4 weeks of the program [54].

#### Assessments before and after the exercise intervention

To ensure that fatigue and dopaminergic medication withdrawal did not influence evaluations of anxiety, assessment with outcome measures took place on two separate days within the pre and post assessment weeks. On the first day of assessment, anxiety, cognition, and quality of life were assessed; all while participants were in the ON medication state. Questionnaires pertaining to anxiety and quality of life were given first to ensure that any potential negative performance on cognitive outcomes did not influence these measures.

On the second day of assessment, patients arrived at the MDRC while OFF dopaminergic medications and were immediately assessed with the UPDRS-III by a movement disorders specialist blinded to group allocation. After which, participants took their normal dopaminergic medication and completed the CHAMPS questionnaire. One hour after taking dopaminergic medications, participants were re-assessed with the UPDRS-III by the same (blinded) movement disorders specialist.

#### Data and statistical analysis

As previously mentioned, participants were not randomized to the non-exercise control group. For this reason, the non-exercise control group was not included in the statistical analysis. However, to serve as a reference, demographics and primary and secondary outcome measure averages and standard deviations of the control group can be found in Table 1 and S1 Table. All results were analyzed using StatSoft STATISTICA 8.0.550. Participants were included in the current study if they completed the primary outcome measure and UPDRS-III in the pre- and post-assessments. Participants that were missing particular secondary measures were excluded from that particular analyses, as their missing data was not filled but left blank.

**Table 1:** Participant characteristics.

	Sensory Attention Focused Exercise	Sham Exercise Control	Non Exercise Control	Between Exercise Group Comparisons
	*M (SD)*	*M (SD)*	*M (SD)*	
Numbers (M/F)	15 (12/3)	15 (12/3)	9 (8/1)	-
Age	73.0 (8.06)	65.4 (6.21)	72.0 (5.52)	t (28) = -2.89, p = 0.007
Weight (Kg)	91.98 (20.45)	78.98 (24.70)	83.57 (13.08)	t (28) = -1.65, p = 0.11
Years Since Diagnosis	6.73 (3.73)	6.60 (5.18)	8.67 (6.02)	t (28) = -0.08, p = 0.94
Levodopa Equivalent Dose	599.38 (369.47)	614.27 (242.53)	901.67 (712.54)	t (28) = -0.13, p = 0.90
UPDRS-III OFF	29.11 (7.02)	29.36 (10.35)	24.88 (10.76)	t (26) = -0.08, p = 0.94
UPDRS-III ON	22.43 (8.63)	20.07 (8.39)	17.61 (10.05)	t (28) = -0.81, p = 0.43
CHAMPS Overall	3936.90 (2353.83)	3204.37 (3392.19)	4313.95 (2586.75)	t (24) = -0.65, p = 0.52
CHAMPS Moderate	2556.83 (2122.21)	1591.92 (2099.14)	2939.87 (2477.97)	t (24) = -1.16, p = 0.26
Percentage Adherence	97.98 (2.47)	96.97 (2.81)	-	t (28) = -1.04, p = 0.30

UPDRS-III = Unified Parkinson’s Disease Rating Scale Motor Section (Sub-section III); OFF = >12 hour withdrawal from dopaminergic medication; ON = 1 hour after taking their normal dopaminergic medication; CHAMPS = Community Health Activities Model Program for Seniors questionnaire; Overall = All physical activity levels; Moderate = Physical activity levels ≥3 METs; Bold numerical font = significant p-value (alpha level was set to p<0.05).

#### Participant demographics

To determine whether age, weight, years since diagnosis, LED, UPDRS-III (both OFF and ON) and CHAMPS (both overall and moderate intensity) were statistically different between the SAFEx and Sham Exercise groups at pre assessment, independent samples t-tests were utilized. Percentage adherence to the exercise program was calculated ([# of classes attended/33] x 100) and an independent samples t-test was used to determine whether exercise groups received a statistically similar number of exercise sessions. Alpha level was set to p<0.05. Since age was different between groups (discussed further in the results), age was included as a covariate variable on all primary and secondary outcome measure analyses described below (ANCOVA).

#### Primary outcome measures

To determine whether participants’ anxiety significantly changed, and to uncover potential differences between groups at pre assessment and post, the State and Trait Anxiety Inventory and PAS questionnaire were subjected to statistical analysis. Responses to the State and Trait Anxiety Inventory were scored separately to reveal a State anxiety score and a Trait anxiety score. Each was subjected to a two-factor mixed repeated measures ANCOVA (2 groups x 2 evaluation times). The PAS comprises three sections that quantify persistent, episodic, and avoidance behaviour anxiety. The total PAS score (including persistent, episodic, and avoidance scores) was first subject to a two-factor mixed repeated measures ANCOVA (2 groups x 2 evaluation times). Subsequently, a two-factor mixed repeated measures ANCOVA (2 groups x 2 evaluation times) was used to assess group differences and changes with respect to each section of the PAS questionnaire.

#### Secondary outcome measures

MOCA scores, Stroop scores (number of responses on the conflict task), Trail-Making-Task times (time to complete parts A and B, and the difference between parts A and B) and Verbal fluency scores (total number of words beginning with F, A, and S) were subjected to two-factor mixed repeated measures ANCOVA analysis (2 groups x 2 evaluation times). Three-factor mixed repeated measures ANCOVA (2 groups x 2 evaluation times x 2 medication states) assessed symptom severity (UPDRS-III) group differences and changes. To investigate whether significant differences in LED, perceived quality of life (PDQ-39) and levels of physical activity (CHAMPS overall and moderate) were present between groups at pre assessment and post; and to determine whether significant changes within groups took place between evaluation time-points, two-factor mixed repeated measures ANCOVA were utilized (2 groups x 2 evaluation times).

#### Post hoc analyses

For the primary outcome measure (PAS), significant interactions were investigated with a Fisher’s LSD test, given this is the first randomized controlled trial aimed to use exercise as a method for anxiety improvement in individuals with Parkinson’s disease [8,55]. We did not adjust for multiple statistical comparisons to ensure that no important findings were missed that could be tested in future randomized controlled trials [56]. Significant interactions that were found for the secondary outcome measures were investigated with a Tukey’s HSD test, which is more stringent.

## Results

Fig 1 presents the flow of participants through the study, including recruitment, randomization, assessment times, and withdraws. No adverse effects related to the PD-SAFEx^™^ exercise programs were reported.

### Participant demographics

Participant demographic effects and p values can be found in Table 1. As stated previously, participants in the SAFEx group were significantly older than the Sham Exercise group (t(28) = -2.89, p = 0.007, *d* = 1.06).

### Primary outcome measures

S1 Table presents all interactions between group and evaluation time with respect to the primary and secondary outcome measures.

**Fig 2:**
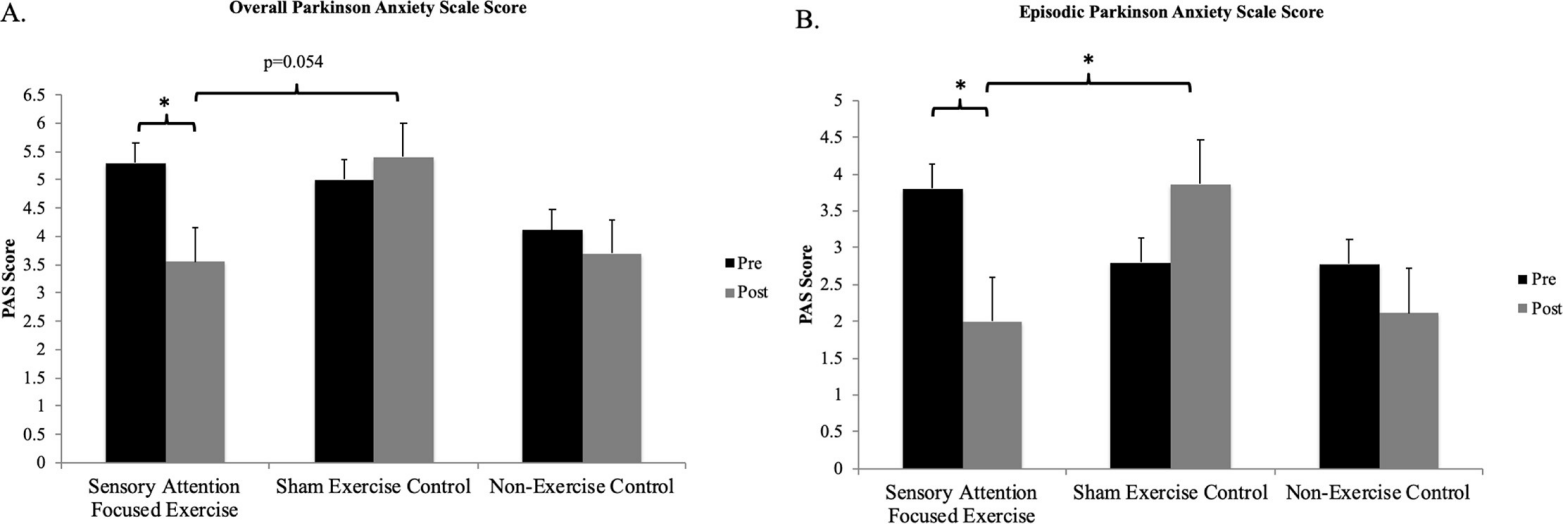
Effect of exercise on anxiety. Reported Overall Parkinson Anxiety Scale (PAS) scores (A.) and Episodic Parkinson Anxiety Scale scores (B.) in participants with Parkinson’s disease before (pre; black) and after (post; grey) 11-weeks of Sensory Attention Focused Exercise, Sham Exercise, or no exercise (non-Exercise Control). Statistical analysis included only exercise groups. * indicates a statistically significant difference p<0.05.

Significant interactions between group and evaluation time were found for the total PAS score (F (1,27) = 5.41, p = 0.028, η_p_^2^ = 0.17) and the episodic section of the PAS questionnaire (F (1,27) = 6.89, p = 0.014, η_p_^2^ = 0.20). Fisher’s post hoc uncovered that from pre assessment to post, overall anxiety (p = 0.07) and episodic anxiety (p = 0.010) scores significantly decreased in the SAFEx group. At post assessment, the SAFEx group’s episodic anxiety scores were significantly lower than the Sham Exercise group’s episodic anxiety scores (p = 0.046) (see Fig 2).

### Secondary outcome measures

S1 Table also presents the significant interaction between group, evaluation time, and medication state for UPDRS-III scores (F (1,25) = 8.79, p = 0.007, η_p_^2^ = 0.26). With respect to the ON medication state, Tukey’s post hoc revealed that UPDRS-III scores significantly decreased in both the SAFEx (p = 0.002) and Sham Exercise groups (p = 0.007). However, in the OFF medication state, UPDRS-III scores significantly decreased in only the Sham Exercise group (p = 0.002). A significant interaction between group and time was found for PDQ-39 (F (1,23) = 5.99,p = 0.022, η_p_^2^ = 0.21), although, Tukey’s post hoc did not reveal any significant differences between groups or evaluation times.

## Discussion

To the best of our knowledge, this is the first randomized controlled trial investigating the effects of exercise on anxiety in individuals with PD. Specifically, we aimed to investigate whether sensory attention focused exercise might improve upon validated measures of anxiety in patients with PD, namely the Parkinson Anxiety Scale (PAS). It was hypothesized that directing patient’s attention towards sensory feedback during a goal-based exercise program may provide benefits to anxiety. Since total anxiety scores and episodic anxiety scores on the PAS were reduced particularly after the Sensory Attention Focused Exercise (SAFEx) intervention but not the Sham intervention, our hypothesis was confirmed. It is important to note that the Sham Exercise control group was well matched to the SAFEx group at baseline (Table 1 and S1 Table; age was included as a covariate in all primary and secondary outcome measure statistical analyses). Furthermore, the Sham Exercise control group completed an identical set of exercises but simply allocated their attention externally rather than internally during the exercise intervention. While improvements in motor symptom severity were found in both exercise groups suggesting that the similar exercises facilitated an improvement in motor function, it was the allocation of attention that determined whether anxiety was influenced by the intervention. At post assessment, the SAFEx group (i.e. those that adopted an internal focus of attention) reported lower levels of total and episodic anxiety than the Sham Exercise group. Since the only difference between groups was the location in which they were instructed to focus attention throughout the exercise program, and only the SAFEx group reported significantly lower anxiety levels, it can be concluded that instructing individuals with PD to focus attention specifically on sensory feedback while completing a goal-based exercise program may provide significant improvements to anxiety. However, by directing patients’ attention away from sensory feedback during exercise, improvements to anxiety may be hindered. A strength of these findings, is that improvements were detected using the PAS questionnaire, which is the most valid measurement of anxiety in individuals with PD to date [44].

**Fig 3:**
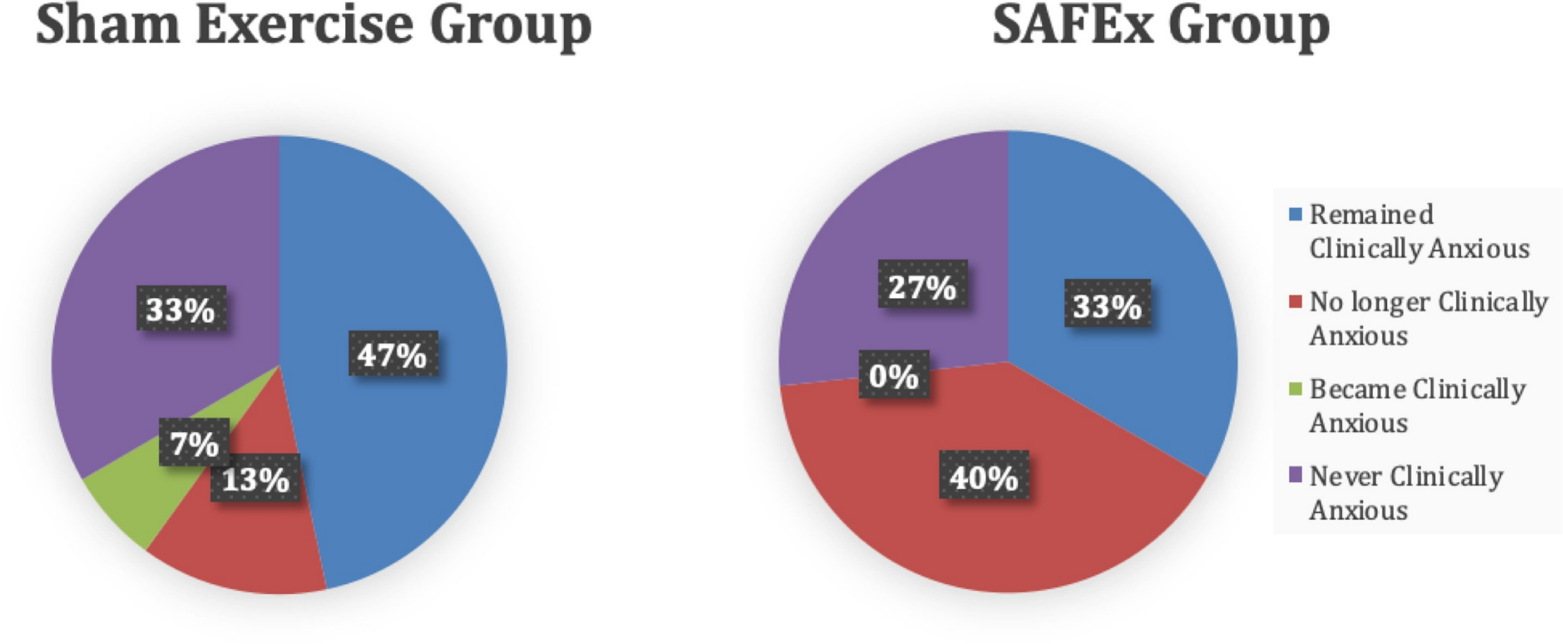
Distribution of clinically anxious participants before and after exercise interventions. Blue–the percentage of each group who had a total PAS score >13 before and after exercise intervention. Red–the percentage of each group who had a total PAS score >13 before the intervention, and <13 after the intervention. Green–the percentage of each group who had a total PAS score of <13 before the intervention, and >13 after the intervention. Purple–the percentage of the group that had a total PAS score of >13 both before and after the intervention.

Prior to the development and validation of the PAS, previous measurements were insufficient to assess anxiety in individuals with PD [44]. Specifically, the Beck Anxiety Inventory (BAI), Hamilton Anxiety Rating Scale (HARS), and the Hospital Anxiety and Depression Scale (HADS) possess poor positive predictive value and only moderate negative predictive value, while lacking construct validity in PD [57]. One reason for these previously mentioned issues might be the lack of distinction between persistent and episodic anxiety in individuals with PD [58]. Persistent anxiety refers to more generalized anxiety with features of major depression, whereas episodic anxiety includes features of panic disorder, social phobia and agoraphobia [58]. These distinct domains (i.e. persistent and episodic anxiety) do not match those in the DSM-IV, and thus standard anxiety scales, such as the BAI, HARS, and HADS, carry bias in measurement for either persistent or episodic anxiety [57]. However, this is not a problem with the PAS, which has recently been validated, and has demonstrated an area under the curve (receiver operating characteristic curve analysis) higher than the BAI, HARS, and HADS [57], making the PAS the current gold standard for measuring anxiety in PD. Overall, since panic disorder, social phobia (i.e. episodic anxiety), and generalized anxiety disorder (i.e. persistent anxiety) have been reported in up to 30%, 50%, and 25% of patients with PD, respectively [59–61], reducing clinical levels of anxiety (PAS total score >13) [44], as has been demonstrated in the present randomized controlled trial (see Fig 3), carries great clinical importance.

Notably, after the exercise intervention, 40% of the SAFEx group were no longer clinically anxious (i.e. pre-PAS total >13, but post-PAS total <13), and no participants in this group progressed to develop clinical levels of anxiety (compared to 10% of participants in the Non-Exercise control group which progressed to develop clinical levels of anxiety). This is in stark contrast to the Sham Exercise group, whereby only 13% of participants in this group were no longer clinically anxious (i.e. pre-PAS total >13, but post-PAS total <13) after the Sham intervention, and 7% of individuals developed clinical levels of anxiety after the intervention (i.e. pre-PAS total <13, but post-PAS total >13) (Fig 3). It is noteworthy that a similar distribution of participants were never clinically anxious in both groups (Sham n = 5; SAFEx n = 4). Taken together, these findings underscore the clinical relevance of this work and demonstrate a clinically meaningful reduction in levels of anxiety in PD patients who received sensory attention focused exercise which can be achieved at low costs and with minimal risk for adverse events.

Whilst there have been few, if any, pharmacological randomized controlled trials with anxiety as the primary outcome in PD, there has been a small number of non-pharmacological studies. A recent study demonstrated that a weekly mindfulness yoga intervention that lasted 8 weeks reduced anxiety in PD (measured by the Hospital Anxiety and Depression Scale), whereas a weekly stretching and resistance exercise intervention did not [62]. Similar to our study, the reduction in anxiety was both statistically and clinically significant. These findings are consistent with the current findings, since an element of the mindfulness yoga intervention included deep breathing and directing attention inward toward body sensations. Another small study of 11 PD patients which implemented an exercise-based behavioural treatment that was focused on increasing movement amplitude (arguably focusing attention on the action outcome rather than internally), did not report improvements in anxiety outcome measures (measured with the Beck Anxiety Inventory) [37]. These findings are also consistent with the null findings from our Sham exercise group. Mixed findings have been found for the effectiveness of Cognitive Behavioral therapy (CBT) for improving anxiety in PD [63]. To the best of our knowledge, no studies to date have investigated the effectiveness of CBT using the PAS, and therefore it remains difficult to directly compare findings. Future research is needed to further investigate whether exercise might be an effective adjunct therapy for anxiety or whether it is necessary to include a cognitive or sensory-attentional component in order to have clinical benefits.

Another important clinical finding from the present randomized controlled trial was improvements to motor symptom severity after the exercise program. Specifically, UPDRS-III scores in the ON state were improved (i.e. decreased) in the SAFEx group from pre assessment to post, while UPDRS-III scores in both the ON and OFF states were improved in the Sham Exercise group. These findings indicate that, regardless of whether participants were instructed to focus attention (i.e. towards sensory feedback (SAFEx) or the movement of coloured labels attached to their limbs (Sham Exercise)), exercise led to a clinically relevant improvement of 4.5 points (in the ON state) as defined by Shulman and colleagues (2010), which is in contrast to the worsening of motor symptoms over time seen in the Non-exercise control group (ON UPDRS-III changes: SAFEx = -4.93; Sham Exercise = -4.67; Non-exercise Control = +1.89) [64]. Secondly, focusing attention towards the effect of an action on the environment during goal-based exercise may provide added benefits to motor symptom severity compared to focusing attention on sensory feedback, since only the Sham Exercise group experienced a clinically relevant improvement to OFF medications UPDRS-III (OFF UPDRS-III changes: SAFEx = -2.22; Sham Exercise = -5.07; Non-exercise Control = +0.87). Interestingly, the 5.07 point decrease in the OFF medications UPDRS-III from pre- to post assessment found in the Sham Exercise group was the only change to surpass a more conservative clinically important change of greater than five points defined by Schrag and colleagues (2006) [65]. These group differences provide further insight into the potential mechanisms underlying the improvements found.

Since OFF medications UPDRS-III assessment may reflect functionality of the basal ganglia without dopamine replacement masking endogenous functioning [66], focusing attention towards the effect of an action on the environment may promote recruitment of circuitries more affected by Parkinson’s pathology (i.e. motor loops of the dorsal striatum that link with the sensorimotor cortex [21], and thus more predisposed to UPDRS-III improvements [20,67–71]. In contrast, recruitment of attentional networks that may compensate for impaired sensory processing that are less affected by Parkinson’s pathology may have been recruited during the SAFEx program, thus not allowing for the same capacity of symptom improvements [20,71]. These speculative conclusions were further supported by the anxiety findings. Since the Sham Exercise group experienced improved symptoms without improved anxiety, unlike the SAFEx group, anxiety experienced by individuals with PD may not be entirely the result of motor symptoms, but may rather have a more pathological origin that was modified by sensory attention focused exercise [1,72–77]. Given the established link between anxiety and movement impairments, we expected that if motor symptoms were modified, so too would anxiety. However, discussion with respect to the pathology underlying anxiety, attention and degeneration in PD may provide insight to this contradiction.

Mesolimbic structures, such as the anterior cingulate cortex, nucleus accumbens and amygdala, have been implicated in anxiety pathology in PD [78–80]. These structures converge at the ventral striatum of the basal ganglia, the same area (i.e. ventral striatum) in which frontal areas involved in attention towards sensory feedback converge [14,21,81]. Neurodegeneration in the ventral striatum has been demonstrated to take place later in PD progression, whereas the dorsal striatum is typically affected earlier [21,82–84]. Therefore, by instructing patients to focus attention internally on sensory feedback during goal-based exercise (SAFEx), functioning of frontal areas involved in attention and emotional processing that bypass the more dorsally affected areas may have specifically improved, providing benefits to anxiety but not OFF medication symptoms [67,71,81]. In contrast, by directing patients to focus on the effect of their actions on the environment (Sham Exercise), more automatic control pathways that project dorsally and are more affected by disease progression may have been recruited [67,71,81], thus providing benefits to OFF medication symptoms, but not influencing anxiety.

A final point of clinical importance pertains to the group by time interaction found for measured quality of life of patients involved in the present study. Although the post hoc did not reveal statistically significant findings, a trend suggested that the SAFEx group reported lower PDQ-39 scores at post assessment compared to pre. Anxiety has been revealed to have the greatest influence on declined quality of life in individuals with PD [29,85,86]. Therefore, it is not surprising that the only exercise group to report lower levels of anxiety (i.e. improved anxiety) after the exercise program was also the only group to report lower scores on the PDQ-39 (i.e. improved quality of life). Since one might argue that improving quality of life should be the end-goal of all interventions in PD, sensory attention focused exercise may be a critical adjunct therapy for improving quality of life in these patients.

The present randomized controlled trial was not without potential limitations. Firstly, since randomization of participants into the non-exercise control group did not take place (in contrast to the exercise groups), statistical comparisons were not made between the exercise groups and the non-exercise control group. Although, since the purpose of the non-exercise control group was only to serve as an important reference point, and the aim of the study did not require a non-exercise control group, we considered this to be a minor limitation. Secondly, the present study did not include a washout period (i.e. a second follow-up assessment taking place after a certain period of time following the post assessment). Washout periods may provide indication as to whether changes to neuronal connections potentially took place after the intervention. With the addition of a washout period, stronger conclusions could have been made regarding the influence of sensory attention focused exercise on anxiety in PD. A final consideration is that the current study made a number of statistical comparisons, and thus some results could have been found by chance. It is reassuring that multiple dimensions of our primary outcome measures (PAS total and PAS episodic) showed similar group effects, providing more confidence in the robustness of our findings. Nonetheless, future research including larger samples is needed to replicate this work.

## Conclusion

Significant improvements to total anxiety and episodic anxiety scores on the Parkinson Anxiety Scale were found in individuals with Parkinson’s disease after 11-weeks of sensory attention focused goal-based exercise. However, Parkinson Anxiety Scale questionnaire scores did not improve in a group of patients with PD who completed an identical exercise program while focusing externally on the effect of their actions on the environment. Notably, motor symptoms while patients were ON medications improved after both exercise interventions. These findings suggest that regardless of where attention is focused during exercise, ON medication symptoms may improve after goal-based exercise. However, instructing individuals with Parkinson’s disease to focus attention specifically on sensory feedback while completing a goal-based exercise program may provide the added benefit of reducing anxiety in PD patients. One explanation for these findings could be that an internal focus on sensory feedback during goal-based exercise may recruit limbic circuitry (e.g. prefrontal cortex, cingulate cortex) involved in attention and top down control of emotional processing, leading to less sensory uncertainty (i.e. reduced conflict signals), which minimizes/regulates the engagement of the amygdala-ventral striatum circuitry, potentially leading to improvements in anxiety. In contrast, a focus on the effect of actions on the environment may recruit more automatic control pathways that project through the dorsal striatum and are more affected by disease, thus providing benefits to symptoms, but not influencing anxiety.

## Supporting information

S1 Checklist CONSORT 2010 checklist of information to include when reporting a randomised trial*. (DOC)

S1 Table Primary and secondary outcome measures. Table displays averages with standard deviations in brackets and statistical analyses comparing pre to post within exercise groups and between groups at pre and post assessment.
(DOCX)

S1 Data (PDF)

S2 Data (DOCX)

S3 Data (XLSX)
