# Correction: Sensory focused exercise improves anxiety in Parkinson’s disease: A randomized controlled trial

**DOI:** 10.1371/journal.pone.0319768

**Published:** 2025-02-25

**Authors:** Eric N. Beck, Mary T. Y. Wang, Brittany N. Intzandt, Quincy J. Almeida, Kaylena A. Ehgoetz Martens

After publication of this article [1], concerns were raised about an error in Fig 2A, which does not align with the data provided in S1 Table. This occurred due to incorrect labeling of the y-axis. The authors have provided a corrected version here. Fig 2 legend is also corrected to clarify that the error bars in the graphs represent standard error of the mean. A member of the *PLOS One* Editorial Board reviewed the updated figure and advised that it supports the results and conclusions reported in the article.

**Fig 2:**
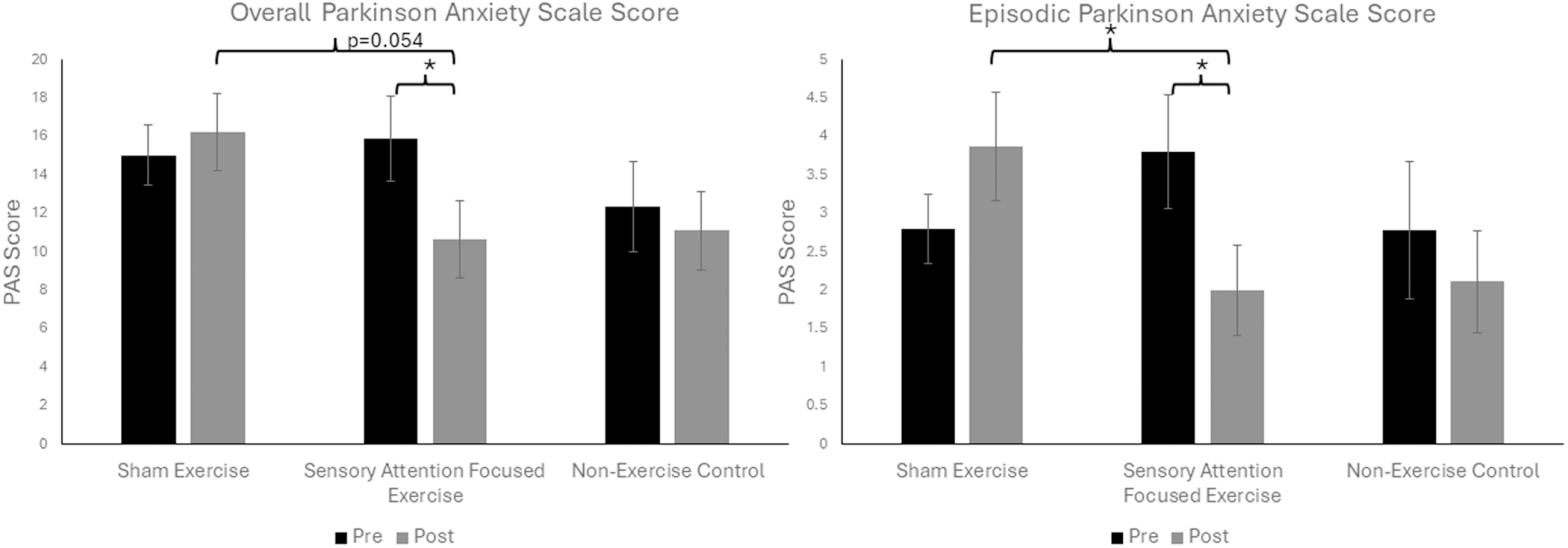
Effect of exercise on anxiety. Reported Overall Parkinson Anxiety Scale (PAS) scores (A) and Episodic Parkinson Anxiety Scale scores (B) in participants with Parkinson’s disease before (pre; black) and after (post; grey) 11-weeks of Sensory Attention Focused Exercise, Sham Exercise, or no exercise (non-Exercise Control). Error bars represent standard error of the mean. Statistical analysis included only exercise groups. * indicates a statistically significant difference p < 0.05.

The authors apologize for the errors in the published article.
