# Supplementary material for: Sensory focused exercise improves anxiety in Parkinson’s disease: A randomized controlled trial
Source: PLoS One. 2020 Apr 16;15(4):e0230803. doi: 10.1371/journal.pone.0230803 (PMC7162490; doi:10.1371/journal.pone.0230803)
Supplement: S1 Table — Table displays averages with standard deviations in brackets and statistical analyses comparing pre to post within exercise groups and between groups at pre and post assessment. (DOCX) [file pone.0230803.s002.docx]

**S1 Table:** **Primary and secondary outcome measures.** Table displays averages with standard deviations in brackets and statistical analyses comparing pre to post within exercise groups and between groups at pre and post assessment.

| Clinical Variables | Sensory Attention Focused Exercise | | | Sham Exercise Control | | | Between Exercise Group Comparison | | Effects (F) |
| --- | --- | --- | --- | --- | --- | --- | --- | --- | --- |
|  | Pre | Post | *p pre-post* | Pre | Post | *p pre-post* | Pre | Post |  |
| PAS  Total | 15.9 (3.9) | 10.7 (3.4) | 0.007 | 15.0 (3.8) | 16.2 (3.9) | 0.514 | 0.758 | 0.054 | F(1,27_)_= 5.41,  p=0.028 |
| PAS  Persistent | 8.5 (4.0) | 6.2 (3.6) | 0.036 | 8.7 (3.6) | 9.0 (3.6) | 0.832 | 0.884 | 0.049 | F(1,27)= 2.64,  p=0.116 |
| PAS  Episodic | 3.8 (2.9) | 2.0 (2.3) | 0.010 | 2.8 (1.7) | 3.9 (2.7) | 0.112 | 0.277 | 0.046 | F(1,27)= 6.89,  p=0.014 |
| PAS  Avoidance | 3.6 (2.8) | 2.5 (2.5) |  | 3.5 (2.4) | 3.4 (2.8) |  | 0.944 | 0.331 | F(1,27)= 0.164,  p=0.211 |
| MOCA | 23.8 (3.4) | 25.7 (3.0) |  | 23.3 (4.8) | 23.6 (5.8) |  | 0.984 | 0.603 | F(1,26)= 1.51,  p=0.230 |
| State Anxiety | 33.2 (11.2) | 33.2 (8.5) |  | 38.8 (12.5) | 39.9 (14.0) |  | 0.191 | 0.267 | F(1,24)= 1.26,  p=0.273 |
| Trait Anxiety | 39.3 (10.7) | 36.2 (10.3) | 0.017 | 38.1 (8.9) | 41.2 (11.4) | 1.0 | 0.756 | 0.451 | F(1,23)= 1.36,  p=0.255 |
| Stroop | 25.3 (5.6) | 25.4 (8.3) |  | 30.8 (10.4) | 30.1 (13.2) |  | 0.667 | 0.991 | F(1,15)= 1.87,  p=0.192 |
| TMT A | 51.7 (18.2) | 48.9 (19.7) |  | 50.6 (32.4) | 72.3 (79.2) |  | 1.0 | 0.955 | F(1,24)= 0.93,  p=0.345 |
| TMT B | 170.0 (129.4) | 123.2 (58.8) |  | 164.7 (176.7) | 213.5 (181.4) |  | 1.0 | 0.647 | F(1,24)= 1.61,  p=0.217 |
| TMT B-A | 118.3 (114.5) | 74.3 (43.1) |  | 114.2 (151.7) | 141.2 (121.9) |  | 1.0 | 0.603 | F(1,24)= 1.51,  p=0.231 |
| Verbal Fluency (FAS) | 34.4 (15.7) | 36.9 (16.4) |  | 27.3 (10.8) | 26.9 (10.2) |  | 0.361 | 0.241 | F(1,25)= 0.21,  p=0.652 |
| UPDRS-III  OFF | 29.11 (7.02)^a^ | 26.89 (8.63)^a^ | 0.464 | 29.36 (10.35)^a^ | 24.29 (8.40)^a^ | 0.002 | 1.0 | 1.0 | F(1,25)= 8.79,  p=0.007 |
| UPDRS-III  ON | 22.43 (8.45) | 17.50 (7.25) | 0.002 | 20.07 (10.76) | 15.40 (7.86) | 0.007 | 1.0 | 1.0 |  |
| LED | 599.4 (369.47) | 596.1 (371.62) |  | 614.3 (242.53) | 605.4 (231.62) |  |  | 1.0 | F(1,27)= 0.15,  p=0.704 |
| PDQ-39 | 39.3 (20.00) | 32.3 (21.49) | 0.060 | 43.7 (28.74) | 45.5 (34.11) | 0.775 | 0.975 | 0.324 | F(1,23)= 5.99,  p=0.022 |
| CHAMPS  Overall | 3936.9 (2353.8) | 5268.3 (4033.4) |  | 3204.4 (3392.2) | 4519.5 (4801.4) |  | 0.981 | 0.923 | F(1,22)< 0.01,  p=0.983 |
| CHAMPS  Moderate | 2556.8 (2122.2) | 2804.9 (2996.5) |  | 1591.9 (2099.1) | 2235.3 (2717.6) |  | 0.827 | 0.867 | F(1,22)= 0.16,  p=0.693 |

**Statistical Analysis was limited to Sensory Attention Focused Exercise and Sham Exercise Control groups. The Non-Exercise Control group was not subject to statistical analysis.** PAS = Parkinson Anxiety Scale; MOCA = Montreal Cognitive Assessment; TMT (A, B, and B-A)= Trail-Making Task Parts A, B, and B-A; UPDRS-III = Unified Parkinson’s Disease Rating Scale Motor Section (Sub-section III); OFF = >12 hour withdrawal from dopaminergic medication; ON = 1 hour after taking their normal dopaminergic medication; LED = Levodopa Equivalent Dose, PDQ-39 = Parkinson’s disease Questionnaire 39; CHAMPS = Community Health Activities Model Program for Seniors questionnaire; Overall = All physical activity levels; Moderate = Physical activity levels $\boldsymbol{\geq}$3 METs; p pre – post = p-value of the change from pre assessment to post; Effects = Group by time interaction or Group by time by medication state interaction (UPDRS-III); ^a^ Signifies a significant difference between UPDRS-III scores ON and OFF meds.
