# Supplementary material for: Sensory focused exercise improves anxiety in Parkinson’s disease: A randomized controlled trial
Source: PLoS One. 2020 Apr 16;15(4):e0230803. doi: 10.1371/journal.pone.0230803 (PMC7162490; doi:10.1371/journal.pone.0230803)
Supplement: S1 Data — (PDF) [file pone.0230803.s003.pdf]

Non-Psychology Applicants - Request for Human Ethics Review

Project Info.

File No: 4542
Project Title: EVALUATING THE UNDERLYING MECHANISM AND INFLUENCE OF SINGLE TASK VS DUAL TASK EXERCISE ON REHABILITATION IN PARKINSON’S DISEASE: A SINGLE BLIND RANDOMIZED CONTROLLED TRIAL
Principal Investigator: Mr. Eric Beck (Faculty of Science\Kinesiology & Physical Education)
Start Date: 2015/06/22
End Date: 2016/10/01
Keywords: Parkinson's disease, sensorimotor integration, sensory, proprioception, automaticity, attention, dual task, exercise, intervention

Project Team Info.

Principal Investigator

Prefix: Mr.
Last Name: Beck
First Name: Eric
Affiliation: Faculty of Science\Kinesiology & Physical Education
Rank: Master's Student
Email: beck3510@mylaurier.ca
Phone1:
Phone2:
Fax:
Primary Address:
Institution: Wilfrid Laurier University
Country: Canada
Comments:

Other Project Team Members

Prefix	Last Name	First Name	Affiliation	Role In Project	Email

	Almeida	Quincy	Faculty of Science\Kinesiology & Physical Education	Supervisor	qalmeida@wlu.ca

Common Questions

1. Project Details

#	Question	Answer
1.1	Which type of researcher are you? (Note: Faculty CAS can only obtain REB clearance while on an active employment contract.)	Student - Masters Program
1.2	If you are a student researcher, is this project for your:	Thesis Research/MRP
1.3	Project Funding: Will this project be supported by research funding?(If 'No' go to question 1.5.)	No
1.4	If 'Yes' to the above question please specifiy the funding source (ex. SSHRC, NSERC etc.) and the grant # or Laurier index code.	

1.5	Provide a succinct summary of the purpose, objectives and aims of the research. Describe your research methodology/design.	Parkinson's disease is a slow progressing neurodegenerative movement disorder with both motor and non-motor symptoms. The gold standard medical pharmacotherapy (levodopa) lends relief from symptoms. However, some of the non-motor symptoms are not responsive to this therapy or eventually become non-responsive, such as impaired proprioception (sensory feedback from the limbs providing awareness of one's body in physical space) (Hamani & Lozano, 2003; Stelmach & Phillips, 1991). Although it is unclear as to why levodopa is unable to relieve proprioception impairments, a key underlying mechanism that may be responsible is an impaired sensorimotor integration (the combination of sensory and motor information in the brain utilized to guide movements) (Conte, Khan, Defazio, Rothwell, & Berardelli, 2013). This is not the result of corrupted input information from proprioceptors into the brain (such as muscle spindles and golgi tendon organs), but rather the downstream integration of this information in the brain. With faulty signalling through the area of the brain damaged in Parkinson's disease (the basal ganglia), deficient sensorimotor integration impairs patients' ability to rely on sensory feedback during movement. This subsequently impairs ability to automatize learned movements in Parkinson's disease and causes previously automatic movements (such as walking) to require conscious control (lose automaticity) (Petzinger et al., 2013). This is problematic in instances where attention is diverted from a movement that is no longer automatically controlled, such as engaging in a conversation while walking. Without attention on the movement, motor control breaks down, leading to heightened

		chance of falls and injury. However, Wulf and Colleagues (2009) demonstrated that when individuals with Parkinson's disease focused externally, and greater reliance on sensorimotor integration was fostered, balance control was improved compared to when these individuals focused attention internally. This suggests that individuals with Parkinson's disease may retain ability to rely on sensorimotor integration. Therefore, by fostering neuroplasticity through the basal ganglia with the utilization of exercise, and improving ability to rely on sensorimotor integration (using dual task training) in individuals with Parkinson's disease, we may be able to improve automaticity of movements, subsequently decreasing the risk of falling and injury. Furthermore, by improving sensorimotor integration, we may improve symptom severity and slow disease progression. In addition to providing improvements to symptoms of Parkinson's disease, this will provide a greater understanding into the pathophysiological mechanisms underlying improvements associated with exercise rehabilitations employed for Parkinson's disease. To date, goal-directed exercises have yet to investigate the rudimentary aspects of the exercise, since no studies have directly compared identical exercise regimens while focusing on task irrelevant information as opposed to task relevant information. For this reason, this study will investigate large magnitude gait and balance training in two groups, one focusing only on the task at hand, and the other focusing on task irrelevant information, such as dual tasks. It is hypothesized that the group of individuals with Parkinson's disease whom participate in the dual tasking exercise rehabilitation

		will show greater improvements to automaticity (dual task gait) and symptom severity than the exercise group that does not perform dual task training. It is further hypothesized that the dual tasking group will show superior improvement in proprioception, finger-tapping performance, and sensory organization (all measures associated with basal ganglia functioning). Study Design: The upcoming exercise intervention methodology will aim to meet the requirements of a 1A silver ranking level, single blind, randomized control trial according to the criterion of the Cochrane Musculoskeletal Review Group (Tugwell, Shea, Simon, Strand, & Wells, 2004). Ninety participants diagnosed with idiopathic Parkinson’s disease will be recruited from the Sun Life Financial Movement Disorders Research and Rehabilitation Centre database at Wilfrid Laurier University (MDRC; Waterloo, Ontario). This present intervention study will span over a 20- week period. Pre-assessments to measure baseline symptoms and functionality will be conducted over the initial 2-week period prior to the start of the intervention. After pre-assessments have been conducted, participants will be randomized to one of three groups. A 12-week exercise program will follow pre-assessment. Each exercise session will last 60 minutes, and will be provided three times per week. Post-assessment of all outcome measures tested prior to the intervention will be assessed in a 2-week period immediately after the cessation of the exercise program. The washout period will last 6 weeks; thereafter assessments will be completed again. Since the present research study aims to understand the mechanism underlying these rehabilitations, as well as

		ensure ecological validity, symptom severity (UPDRS-III) will be assessed in all individuals in both the “ON” and “OFF” state of dopaminergic medication. Methods of collecting data “ON” and “OFF” dopaminergic medication state will follow our previously constructed standard operating procedures (SOP#3549). Since the individuals are in the “ON” state during all daily activities, exercise classes will be performed in the “ON” dopaminergic medication state. If there are individuals who are naïve to dopaminergic medication (De novo), they will only be assessed once (in the “OFF” dopaminergic state). All individuals included in the rehabilitation program will be required to complete a Physical Activity Readiness Medical Examination (ParMed X), signed by a physician prior to joining the rehabilitation program. Pre/Post/Washout Testing:Task 1: Unified Parkinson’s Disease Rating Scale Part III (Motor Section): Dr. Quincy Almeida, a movement disorders specialist blinded to group assignment, will administer this assessment. The UPDRS-III is a scored examination indicative of motor symptom severity. Task 2: Measurement of Proprioception (Gliding Sled): Utilizing the method designed by Maschke et al. (2003) to measure threshold or limb movement perception, participants will be instructed to inform the tester as to whether the apparatus in which participants will be utilizing moves towards their body, away from their body, or if they are not sure. This protocol will provide a direct measure of proprioceptive threshold, and therefore, perception of sensory feedback, indicative of sensorimotor integration. Task 3: Sensory Organization Protocol (SOP): Following the protocol employed by Nashner and Peters (1990),

		postural stability will be measured in six conditions utilizing the Biodex Balance System™ SD. Center of pressure displacement will be the primary measure of postural stability. This protocol will create sensory feedback conflict, and improved postural stability would be indicative of improved sensorimotor integration. Task 4: Tapping Task: Impaired simple finger tapping amplitude and variability is improved with dopaminergic replacement in individuals with Parkinson's disease, suggesting that tapping is a strong indication of basal ganglia modulation of motor performance for tapping tasks (Harrington et al., 1998; Merchant et al., 2008). Additionally, Elsinger et al. (2003) found that individuals with Parkinson's disease demonstrated reduced sensorimotor cortex activity compared to healthy individuals when performing tapping tasks, indicating the importance of sensorimotor integration for tapping. Therefore, by utilizing the methods employed by Yahalom et al. (2004), an objective measure of basal ganglia function before and after the intervention can be assessed. Task 5: Single Task and Dual Task Walking: Participants will be asked to walk along a 9.75m long and 0.61m wide ProtoKinetics Movement Analysis Software™ electronic walkway carpet (Zeno Walkway – ProtoKinetics, Havertown, PA, USA) at their self pace while either performing a secondary task in 5 trials and not performing a secondary task in another 5 trials. The secondary task, or dual task, will be a phoneme-monitoring task in which participants will count numbers announced by an audio-track. This task will provide a measure of automaticity to provide insight into whether the proposed intervention was capable of

		promoting improvements to automaticity of walking. Task 6: Measures of Executive Function: Participants will be asked to complete four pen-and-paper/interview /questionnaire tasks with the investigator. The first is the Stroop task to provide a validated measure inhibition. The second measure of executive function that will be completed is the Trail-Making-Task for quantification of set-shifting, or the ability to adjust attention in response to changing goals and environmental stimuli. The third measure of executive function will include the digit-span test to assess working memory, or the ability to cognitively hold and process temporally relevant stimuli. Last, to measure general cognitive status, the Montreal Cognitive Assessment will be completed. Task 7: Parkinson's disease Questionnaire 39 (PDQ-39) – A validated questionnaire for individuals with Parkinson's disease to address well-being and perceived quality of life.Exercise Intervention:After assessment with the outcome measures (above) prior to the beginning of the intervention, sixty of the 90 individuals with Parkinson's disease will be recruited from a list of participants in the database who have expressed interest in participating in rehabilitation studies at the centre and thirty individuals with Parkinson's disease who have not expressed the same interest will also be recruited. The sixty participants interested in rehabilitation will then be randomized (through computer program) to one of two groups (30 individuals per group); either the Parkinson's disease Sensory Attention Focused Exercise (PD-SAFEx™) group (focus on task relevant information) or the PD-SAFEx™ + Dual tasking group (focus on task irrelevant information, secondary tasks). The thirty individuals recruited that

		are not interested in committing to the exercise rehabilitation will be assigned to the control group, and will be asked not to change aspects of their daily lives. To determine whether the control group changes in physical activity level throughout the duration of the present study, the Community Health Activities Model Program for Seniors questionnaire (CHAMPS questionnaire) will be utilized. This is a pen-and-paper questionnaire that the participants in the control group will be asked to complete. After randomization, the primary investigator will check for homogeneity between groups with respect to the demographics and symptom severity outcome measures assessed prior to the intervention. If groups are matched, we will proceed with interventions that will be discussed next. If groups are not matched, randomization procedures will be conducted again until homogeneity between groups is achieved. All subjective assessments (UPDRS-III) measured before and after the intervention period by Dr. Quincy Almeida, a movement disorders specialist, blinded to group assignment. The gait, balance, stretching and coordination exercises that will be provided in the proposed intervention to the two exercise groups will follow the exact Parkinson's disease Sensory Attention Focused Exercise (PD-SAFEx™) protocol designed by Sage and Almeida (2009). PD-SAFEx™ is a group setting intervention, which will be led by the primary investigator who is trained in conducting the exercise protocol. The first half of the exercise class will include walking exercises that include large magnitude and coordinated movements. All walking will be performed slowly. Walking exercises will be followed by balance,

		stretching, and coordination exercises while sitting in, or standing near, standard office chairs, utilizing latex Thera-bands®. To ensure that balance and coordination are constantly challenged throughout the intervention, the exercise program progresses each week, increasing in difficulty. Depending on the task, the first set of each exercise will be performed with 'eyes-open' to familiarize participants with the task, and subsequent sets will be performed with 'eyes-closed'. If there is only one set for a specific exercise, the first half of the repetitions will be performed with 'eyes-open' and the second half with 'eyes-closed'. Specific details regarding exercises performed, the numbers of sets and repetitions to be performed, as well as verbal instructions are provided in the Attachment Section under the "PD-SAFEx Weekly Exercise" attachment. The PD-SAFEx™ protocol will be provided to both exercise groups. Below are the protocol differences between groups. Group 1: Original PD-SAFEx™While performing the exercises in PD-SAFEx™, participants will be instructed to focus their attention on sensory feedback. This will include focusing participants' attention on the stretch in their limbs while walking, on the straightness of their backs while sitting, on limb and body orientation in space while coordinating their movements, and on chest movements during breathing exercises. Throughout each exercise session, the instructor and volunteers will constantly provide attention-directing instructions.3. BESIDES PARTICIPATING IN THIS EXERCISE PROGRAM, THIS GROUP WILL BE ASKED TO REFRAIN FROM CHANGING ACTIVITIES OF THEIR DAILY LIVES THROUGHOUT THE 20-WEEK DURATION OF THE

		EXPERIMENT (FROM PRE-ASSESSMENT TO WASHOUT). Group 2: Dual Tasking PD-SAFEx™While performing the exercises from the PD-SAFEx™ program, participants will be instructed to focus their attention on the performance of a secondary task, and not sensory feedback. Participants will be reminded and encouraged by the exercise instructor and volunteers to perform all exercises while focusing attention on the secondary task. 2. THE DUAL TASKS THAT WILL BE COMPLETED WHILE THE PARTICIPANTS PERFORM WALKING, BALANCE, AND/PR SEATED EXERCISES ARE DESCRIBED IN THE “DUAL TASKS TO BE EMPLOYED” ATTACHMENT, FOUND IN THE ATTACHMENT SECTION. The dual tasks that will be performed while the participants perform walking, balance, and/or seated exercises are described in the “dual tasks to be employed” attachment in the attachment section. All dual tasks incorporated into the exercise program will not be performed during each session, but rather the tasks will vary from week-to-week, and difficulty of tasks will be increased with progression through the exercise program. 3. BESIDES PARTICIPATING IN THIS EXERCISE PROGRAM, THIS GROUP WILL BE ASKED TO REFRAIN FROM CHANGING ACTIVITIES OF THEIR DAILY LIVES THROUGHOUT THE 20-WEEK DURATION OF THE EXPERIMENT (FROM PRE-ASSESSMENT TO WASHOUT).Group 3: Control GroupThis group will be asked to refrain from changing activities of their daily lives throughout the 20-week duration of the experiment (from pre-assessment to washout).

1.6	Outline the specific procedures or activities involving the human participants. Exactly what will the participants be asked to do?	Study Design: Ninety participants diagnosed with idiopathic Parkinson's disease will be recruited from the Sun Life Financial Movement Disorders Research and Rehabilitation Centre database at Wilfrid Laurier University (MDRC; Waterloo, Ontario). This present intervention study will span over a 20- week period. Pre-assessments to measure baseline symptoms and functionality will be conducted over the initial 2-week period prior to the start of the intervention. After pre-assessments have been conducted, participants will be randomized to one of three groups. A 12-week exercise program will follow pre-assessment. Each exercise session will last 60 minutes, and will be provided three times per week. Post-assessment of all outcome measures tested prior to the intervention will be assessed in a 2-week period immediately after the cessation of the exercise program. The washout period will last 6 weeks; thereafter assessments will be completed again. Each pre-, post-, and washout assessment will require approximately 130 – 160 minutes. Since the present research study aims to understand the mechanism underlying these rehabilitations, as well as ensure ecological validity, symptom severity (UPDRS-III) will be assessed in all individuals in both the "ON" and "OFF" state of dopaminergic medication. Methods of collecting data "ON" and "OFF" dopaminergic medication state will follow our previously constructed standard operating procedures (SOP#3549). Therefore, each participant that agrees to participate in the "ON" and "OFF" aspect of the study (those who do not agree will still be able to participate in the study and testing will include all assessments, but only the ON assessment will take place)

		will complete all outcome measure assessments twice for the pre-test (ON and OFF meds), twice at post-test (ON and OFF meds), and twice at the washout (ON and OFF meds). Since the individuals are in the “ON” state during all daily activities, exercise classes will be performed in the “ON” dopaminergic medication state. If there are individuals who are naïve to dopaminergic medication (De novo), they will only be assessed once (in the “OFF” dopaminergic state). All individuals included in the rehabilitation program will be required to complete a Physical Activity Readiness Medical Examination (ParMed X), signed by a physician prior to joining the rehabilitation program.<br>Pre/Post/Washout Testing:Task 1: Unified Parkinson’s Disease Rating Scale Part III (Motor Section): Dr. Quincy Almeida, a movement disorders specialist blinded to group assignment, will administer this assessment. The UPDRS-III is a scored examination indicative of motor symptom severity. Task 2: Measurement of Proprioception (Gliding Sled): Utilizing the method designed by Maschke et al. (2003), participants will be asked to sit straight beside the apparatus with their right or left arm (will measure both due to unilateral disease progression) abducted 900, forearm pronated, with 900 flexion at the elbow. The participants’ arm will rest on and strapped to a plastic forearm support (8cm wide by 50 cm long). At the end of the forearm support closest the participants’ elbow, an axel bearing mounted to the table and the forearm support will allow flexion and extension movements at the elbow. At the end of the forearm support closest to the participants’ fingers, an aluminum rod attached to the bottom of the forearm support will connect

		to an aluminum-gliding sled mounted to the table that the apparatus is placed on. A torque motor will be utilized to move the forearm support along the sled at a velocity of 0.50/s (acceleration < 0.00/s). Participants will be blindfolded and wearing headphones while the apparatus moves their forearm at the elbow by 0.2, 0.6, 1.0, 2.0, 3.0, 4.0, 5.0, 6.0, 7.0, or 8.0 degrees. Participants will be instructed to inform the tester as to whether the apparatus moved towards their body, away from their body, or if they are not sure. Electromyography electrodes will collect muscle activity from the biceps and triceps muscles to determine whether the participants make active movements. In the event that this occurs, the trial will be excluded from analysis. This protocol will provide a direct measure of proprioceptive threshold, and therefore, perception of sensory feedback, indicative of sensorimotor integration. Task 3: Sensory Organization Protocol (SOP): Following the protocol employed by Nashner and Peters (1990), postural stability will be measured in six conditions utilizing the Biodex Balance System™ SD. Center of pressure displacement will be the primary measure of postural stability. Participants will be asked to stand quietly on the Biodex Balance System™ SD platform, approximately 1-metre from the wall with their arms at the side. This protocol will create sensory feedback conflict, and improved postural stability would be indicative of improved sensorimotor integration. The conditions are as follows:a.Baseline (SOP #1) – Eyes open, solid surface (somatosensory, visual, and vestibular feedback all accurate)b.SOP #2 – Eyes closed, solid surface (somatosensory and vestibular feedback accurate, visual feedback removed)c.SOP

		#3 – Eyes open with opaque/translucent goggles, solid surface (somatosensory and vestibular feedback accurate, visual feedback inaccurate)d.SOP #4) – Eyes open, foam surface (visual, and vestibular feedback accurate, somatosensory feedback inaccurate)e.SOP #5 – Eyes closed, foam surface (vestibular feedback accurate, visual feedback removed, somatosensory feedback inaccurate)f.SOP #6 – Eyes open with opaque/translucent goggles, foam surface (vestibular feedback accurate, somatosensory and visual feedback inaccurate)Task 4: Tapping Task: Impaired simple finger tapping amplitude and variability is improved with dopaminergic replacement in individuals with Parkinson's disease, suggesting that tapping is a strong indication of basal ganglia modulation of motor performance for tapping tasks (Harrington et al., 1998; Merchant et al., 2008). Additionally, Elsinger et al. (2003) found that individuals with Parkinson's disease demonstrated reduced sensorimotor cortex activity compared to healthy individuals when performing tapping tasks, indicating the importance of sensorimotor integration for tapping. Therefore, by utilizing the methods employed by Yahalom et al. (2004), an objective measure of basal ganglia function before and after the intervention can be assessed. The participants' digit tapping spatiotemporal characteristics (amplitude, velocity, and timing variability) will be tracked by one horizontally oriented Optotrak® camera (Northern Digital, NDI, Waterloo, Ontario). Participants will be asked to complete four tapping conditions:a.Tapping at a comfortable paceb.Tapping as quickly as possiblec.Tapping with a metronome frequencyd.Tapping synchronized with a

		metronome, followed by continuation of that tapping frequency without the metronome Task 5: Single Task and Dual Task Walking: Participants will be asked to walk along a 9.75m long and 0.61m wide ProtoKinetics Movement Analysis Software™ electronic walkway carpet (Zeno Walkway – ProtoKinetics, Havertown, PA, USA). This electronic walkway will measure spatiotemporal characteristics of the individuals' gait while they walk with and without the performance of a dual task. Specifically, we will measure: i) step length, ii) step time, iii) double support time percentage, iv) velocity, v) step length variability, vi) step time variability, and vii) double support time percentage variability. The dual task that will be performed by the participants with Parkinson's disease will be a phoneme monitoring dual task not trained in the intervention. Participants will walk along the Zeno Walkway while listening to an audio track announcing numbers in random order, greater than zero and less than ten. Prior to walking, participants will be assigned two digits and instructed to silently count (without use of fingers) the number of times those two digits were announced by the audio track, separately. The order that digits will be presented throughout the audio track will be randomized in each trial of dual tasking, and the instructed numbers to count will differ between trials. The audio track will present digits with a randomized auditory inter-stimulus interval, varying from 100ms to 1000ms, in order to prevent gait synchronization with the audio track. Once participants have started to walk, they will be asked to continue counting the digits for the entire 12-second duration of the audio track, even if they have finished walking.

		After the trial, participants will be asked to inform the tester of the number of times they heard the two digits they were instructed to count. 2. FOR EXAMPLE, IF THE AUDIO TRACK ANNOUNCED THREE 1'S, TWO 2'S, FOUR 4'S, THREE 5'S, TWO 7'S, AND ONE 9, THE PARTICIPANT WILL BE ASKED TO INFORM THE INVESTIGATOR OF THE AMOUNT OF TIMES THEY HEARD THE AUDIOTRACK ANNOUNCE THE NUMBER 2 AND THE NUMBER 4. This will be recorded and the difference between the participant's response and the actual number of digits will be calculated to determine the error. This will help distinguish if participants allocate attention to the digit-monitoring task or to walking. Before any walking trials are performed, a seated baseline trial of dual task performance will be assessed. This will allow us to quantify the degree to which gait interferes with the dual task.Task 6: Measures of Executive Function: Participants will be asked to complete four pen-and-paper/interview /questionnaire tasks with the investigator. The first is the Stroop task to provide a validated measure of ability to inhibit a dominant, overlearned response. The second measure of executive function that will be completed is the Trail-Making-Task for quantification of set-shifting, or the ability to adjust attention in response to changing goals and environmental stimuli. The third measure of executive function will include the digit-span test to assess working memory, or the ability to cognitively hold and process temporally relevant stimuli. Last, to measure general cognitive status, the Montreal Cognitive Assessment will be completed. Task 7: Parkinson's disease Questionnaire 39 (PDQ-39) – Participants

		will be asked to complete a pen-and-pencil validated questionnaire for individuals with Parkinson’s disease to address well-being and perceived quality of life.Exercise Intervention:After assessment with the outcome measures (above) prior to the beginning of the intervention, sixty of the 90 individuals with Parkinson’s disease will be recruited from a list of participants in the database who have expressed interest in participating in rehabilitation studies at the centre and thirty individuals with Parkinson’s disease who have not expressed the same interest will also be recruited. The sixty participants interested in rehabilitation will then be randomized (through computer program) to one of two groups (30 individuals per group); either the Parkinson’s disease Sensory Attention Focused Exercise (PD-SAFEx™) group (focus on task relevant information) or the PD-SAFEx™ + Dual tasking group (focus on task irrelevant information, secondary tasks). The thirty individuals recruited that are not interested in committing to the exercise rehabilitation will be assigned to the control group, and will be asked not to change aspects of their daily lives. To determine whether the control group changes in physical activity level throughout the duration of the present study, the Community Health Activities Model Program for Seniors questionnaire (CHAMPS questionnaire) will be utilized. This is a pen-and-paper questionnaire that the participants in the control group will be asked to complete. After randomization, the primary investigator will check for homogeneity between groups with respect to the demographics and symptom severity outcome measures assessed prior to the intervention. If groups are matched, we will proceed with interventions that will be

		discussed next. If groups are not matched, randomization procedures will be conducted again until homogeneity between groups is achieved. All subjective assessments (UPDRS-III) measured before and after the intervention period by Dr. Quincy Almeida, a movement disorders specialist, blinded to group assignment. The gait, balance, stretching and coordination exercises that will be provided in the proposed intervention to the two exercise groups will follow the exact Parkinson's disease Sensory Attention Focused Exercise (PD-SAFEx™) protocol designed by Sage and Almeida (2009). PD-SAFEx™ is a group setting intervention, which will be led by the primary investigator who is trained in conducting the exercise protocol. The first half of the exercise class will include walking exercises that include large magnitude and coordinated movements. All walking will be performed slowly. Walking exercises will be followed by balance, stretching, and coordination exercises while sitting in, or standing near, standard office chairs, utilizing latex Thera-bands®. To ensure that balance and coordination are constantly challenged throughout the intervention, the exercise program progresses each week, increasing in difficulty. Depending on the task, the first set of each exercise will be performed with 'eyes-open' to familiarize participants with the task, and subsequent sets will be performed with 'eyes-closed'. If there is only one set for a specific exercise, the first half of the repetitions will be performed with 'eyes-open' and the second half with 'eyes-closed'. Specific details regarding exercises performed, the numbers of sets and repetitions to be performed, as well as verbal instructions are provided in the

		Attachment Section under the “PD-SAFEx Weekly Exercise” attachment. The PD-SAFEx™ protocol will be provided to both exercise groups. Below are the protocol differences between groups. Group 1: Original PD-SAFEx™While performing the exercises in PD-SAFEx™, participants will be instructed to focus their attention on sensory feedback. This will include focusing participants’ attention on the stretch in their limbs while walking, on the straightness of their backs while sitting, on limb and body orientation in space while coordinating their movements, and on chest movements during breathing exercises. Throughout each exercise session, the instructor and volunteers will constantly provide attention-directing instructions. Group 2: Dual Tasking PD-SAFEx™While performing the exercises from the PD-SAFEx™ program, participants will be instructed to focus their attention on the performance of a secondary task, and not sensory feedback. Participants will be reminded and encouraged by the exercise instructor and volunteers to perform all exercises while focusing attention on the secondary task. The dual tasks that will be performed while the participants perform walking, balance, and/or seated exercises are described in the “dual tasks to be employed” attachment in the attachment section. All dual tasks incorporated into the exercise program will not be performed during each session, but rather the tasks will vary from week-to-week, and difficulty of tasks will be increased with progression through the exercise program.Group 3: Control GroupThis group will be asked to refrain from changing activities of their daily lives throughout the 20-week duration of the experiment (from pre-assessment to

		washout).

1.7	How long will it take for the participants to complete the procedures or activities?	The participants with PD will be asked to participate in two experimental testing sessions at each testing period (pre-12 week intervention, post-12 week intervention, washout period 6 weeks after intervention), ON and OFF dopaminergic medication state. Each experimental testing duration in which the participant's visit to the lab will be approximately 130-160 minutes: 45 minutes to complete the UPDRS-III (task 1), 20 minutes to complete the measurement of proprioception (task 2), 20 minutes to complete the sensory organization protocol (task 3), 20 minutes to complete the tapping task (task 4), 10 minutes to perform the ten single task/ dual task walking trials (task 5), 20 minutes to complete the four measures of executive function (task 6), and 10 minutes to complete the Parkinson's disease Questionnaire 39 (task 7). Therefore, in total, participants will be asked to complete a maximum of 780 – 960 minutes of experimental testing in the 20-week period of the study (260 at pre-intervention [ON and OFF], 260 at post-intervention [ON and OFF], and 260 at washout [ON and OFF]). The exercise program will take place over a 12-week period, with three exercise classes a week, each lasting 60 minutes. Therefore, participants will be asked to complete 2160 minutes of exercise. Thus, with the combination of experimental testing and completing the exercise program, participants will be asked to dedicate approximately 2940 – 3120 minutes to the research study. The supervisor, professor Almeida, will conduct the UPDRS-III assessment. However, professor Almeida will not be present during any of the other experimental testing procedures (task 2-11). Therefore, professor Almeida will not be present

		during the experimental collection of measures for executive function. Although, professor Almeida has been clinically trained to conduct these cognitive assessments and has been effectively trained the primary investigator, Eric Beck to carry out these assessments. Furthermore, the primary investigator has two years experience with these measures of executive function, as they were utilized in a previous undergraduate thesis, as well as previous summer projects.
1.8	Will participants be asked to repeat this or any other procedure at a future date as a result of participating in this project? (If 'No' go to question 1.10.)	Yes
1.9	If you marked 'Yes' to the above question provide your response here:	As previously stated, participants will be asked to complete 6 experimental sessions (pre, post, washout in both ON and OFF medication states). Furthermore, Participants that are randomized to the intervention groups will be asked to attend 36 sessions of exercise over a 12-week period (3x/week).
1.10	Who will be collecting the data from participants?	Eric Beck will be the primary investigator, collecting experimental data from the participants. Professor Almeida will collect UPDRS data from participants with Parkinson's disease.

1.11	How will the data be collected and recorded? (ex. hand written notes, video recording etc.)	Task 1: Unified Parkinson's Disease Rating Scale Part III (Motor Section): Data will be collected by Dr. Quincy Almeida and recorded by paper-and-pencil, then transferred to an excel file. Task 2: Measurement of Proprioception (Gliding Sled): Data regarding whether participants perceived a movement towards them, away from them, or whether participants were unsure will be recorded via paper-and-pencil, which will then be transferred to an excel file. Task 3: Sensory Organization Protocol (SOP): Postural stability will be measured utilizing the Biodex Balance System™ SD. Data will be recorded using paper-and-pencil methods and transferred to excel files. Task 4: Tapping Task: The participants' digit tapping spatiotemporal characteristics (amplitude, velocity, and timing variability) will be tracked by one horizontally oriented Optotrak® camera (Northern Digital, NDI, Waterloo, Ontario). This data will be analyzed by the MATLAB® processing engine, and transferred to excel files.Task 5: Single Task and Dual Task Walking: Walking data will be collected along a 9.75m long and 0.61m wide ProtoKinetics Movement Analysis Software™ electronic walkway carpet (Zeno Walkway – ProtoKinetics, Havertown, PA, USA). This data will be transferred to excel files. Tasks 6 and 7: Measures of Executive Function and PDQ-39: All questionnaire outcomes are recorded via paper-and-pencil, and total outcome scores will be transferred to excel files.

2. Clinical & Enhanced Research Methods

#	Question	Answer

2.1	Does this study involve an investigation with participants that evaluates the effects of one or more health-related interventions on health outcomes?	Yes
2.2	Does this study involve research that involves the evaluation of an intervention or experimental therapy, usually by comparing 2 or more approaches (e.g., process-of-care changes, preventive care, manual therapies, psychotherapies)?	Yes
2.3	Does this study involve the use or administration of any health products, drugs, medical devices or biological matter (e.g., radiopharmaceuticals, biological products, medical procedures, cells, genetic therapies)?	No
2.4	If you answered 'Yes' to any of the above questions, this project fits the definition of a clinical trial as defined in TCPS2. As a result this study needs to be registered as a clinical trial (per section 11.3 in TCPS2) in an easily web accessible public registry before you begin to collect data from any participants.Please register your trial with one of the registries listed below in Point 2.5.Forward the registration number to the REB as it is required as part of the REB approval process	
2.5	A) www.anzctr.org.au B) www.clinicaltrials.gov C) www.ISRCTN.org D) https://www.umin.ac.jp/ctr/index.htmE) www.trialregister.nl F) https://eudract.ema.europa.eu/	
2.6	Note: Publishers are increasingly refusing to publish clinical research that has not been registered. Registration cannot take place retroactively.	

2.7	Registration is also beneficial as Health Canada notes that failing to publicly disclose information on a clinical trial can:A) Reduce efficiency in researchB) Result in the suppression of negative resultsC) Prevent prospective participants from becoming involved, andD) Prevent the public from assessing the safety and effectiveness of a trial - information that is necessary for participants to have in order to make informed choices	
2.8	Does this project involve communicating to a participant (or his or her) personal representative a diagnosis identifying a disease or disorder as the cause of their symptoms upon which they are likely to rely on?	No
2.9	Does this project involve performing a procedure on tissue below the dermis?	No
2.10	Does this project involve administering a substance by injection or inhalation?	No
2.11	Does this project involve an application of an electrical, thermal or magnetic modality to a human participant (e.g., MRI, TMS, electrical stimulation, heat, ultrasound, ice etc.)?	No
2.12	Does this project involve treating by means of psychotherapy technique, delivered through a therapeutic relationship, an individual's serious disorder of thought, cognition, mood, emotional regulation that may seriously impair the individual's judgement, insight, behavior, communication or social functioning?	No
2.13	Does this project involve the use of equipment / procedures that requires sterilization?	No

3. Participant Recruitment

#	Question	Answer
3.1	How many participants will be involved in this study?	Ninety participants diagnosed with idiopathic Parkinson’s disease will be involved in this study.

3.2	Describe the potential participants in this research indicating gender (e.g., trans, genderqueer, gender non-conforming), age range, location, and any other special characteristics.	Inclusion Criteria•Either gender, no age or location restriction •Diagnosed with idiopathic PD by a Neurologist•Able to stand 2 minutes, unassisted •Able to walk 10 meters, unassisted •Able to understand English instructionsExclusion Criteria•A neurological disease other than PD•Peripheral neuropathy •Clinically diagnosed with dementia (as stated in the patient's information chart from the patient database at the Sun Life Financial Movement Disorders Research and Rehabilitation)
3.3	How and by whom will the prospective participants be identified?	Each participant with PD will be recruited from the Sun Life Financial Movement Disorders Research and Rehabilitation Centre (MDRC) patient database. All participants will be identified by their participant code in their file.
3.4	How and by whom will they be invited to participate? (Attach a copy of any advertisement, poster or letter used for recruitment using the 'Attachments' Tab above. Even if another person or agency is doing the recruitment, you must provide the REB with a copy of all recruitment materials).	The primary investigator will contact participants by phone call or direct conversation to invite them to participate in the present study.

4. Free and Informed Consent

#	Question	Answer
4.1	How will informed consent be obtained? You must attach a copy of your consent form/information letter using the 'Attachments' Tab above. (Click on the blue 'i' for information on how to prepare this document.)	Participants will read and sign an informed consent letter prior to participating in the study.
4.2	A signed consent form is often used, but there are situations when it is not required or appropriate. If a signed consent form is not being used, please explain why it is not appropriate.	Not Applicable

4.3	Does the study's design require that information about the participants be sought from a third party or any other source (e.g. employer, case worker, family member, teacher, official records or files)? (If 'No' go to question 4.5.)	No
4.4	If you marked 'Yes' to the above question provide your response here:	
4.5	Do the proposed participants in your study include captive, dependent, or vulnerable persons? (If 'No' go to question 4.7.)	No
4.6	If you marked 'Yes' to the above question provide your response here:	
4.7	Do the proposed participants consist primarily of persons from cultures, countries, or ethnic groups different from those of the investigator(s)? (If 'No' go to the next tab 'Research with Children').	No
4.8	If you marked 'Yes' to the above question provide your response here:	

5. Research Involving Children

#	Question	Answer
5.1	Do the proposed participants in your study include persons under the age of 16? Normally, persons age 16 and over may give effective consent on their own behalf. (If 'No' proceed to the next tab above "Risks to Participants.")	No
5.2	How and by whom will the children be asked to participate in your study?	
5.3	What measures will be taken to ensure that the children understand the research and their participation in it?	
5.4	Does this project involve students enrolled in the local school boards (WRDSB and/or WCDSB)?	No

6. Risks to Participants

#	Question	Answer
6.1	Are there any physical risks regarding this research (e.g., exercise leading to muscle damage)? (If 'No' go to question 6.3.)	Yes

6.2	If you marked 'Yes' to the above question provide your response here:	Pre-, Post-, and Washout Tests•Participants may become fatigued due to the number of tasks and trials•Participants may feel unsteady and unsafe while completing the walking and balancing conditions•Since participants will be asked to stand on the Biodex Balance System SD in conditions where sensory information is manipulated, there is an increased chance of participants becoming unbalanced and falling. For this reason, while the participants stand quietly, a spotter will stand directly behind the participant, ready to lend support at any time that the participant requires assistance or becomes unstable. While participants walk along the Zeno Walkway during the single and dual task walking trials, a spotter will walk beside the participant, again ready to lend support at any time that the participant requires assistance or becomes unstable. •For all other data collection procedures (Besides the UPDRS-III), participants will remain seated, with little risk of injury.•5. THERE ARE NO KNOWN RISKS OF DELAYING MEDICATION (TYPICALLY NO LONGER THAN 14 HOURS), SINCE IT WOULD BE SIMILAR TO HOW PARTICIPANTS WITH PARKINSON’S DISEASE SLEEP THROUGH THE NIGHT WITHOUT TAKING ANY ANTI-PARKINSON’S MEDICATION. DELAYING MEDICATION MIGHT ALSO FEEL SIMILAR TO HOW INDIVIDUALS WITH PARKINSON’S DISEASE MIGHT FEEL IF THEY WERE TO HAVE FORGOTTEN TO TAKE A DOSAGE OF THEIR MEDICATION DURING THE DAY. IN SOME CASES, PARTICIPANTS MAY NOT NOTICE ANY DIFFERENCES AT ALL. ALTHOUGH THIS MEDICATION DELAYING PROCEDURE DOES NOT NEED TO BE

		APPROVED BY A PHYSICIAN PRIOR TO THE EXPERIMENT, IF THE PARTICIPANTS HAVE ANY QUESTIONS WHATSOVER REGARDING THE RISKS OF THIS DELAYING PROCEDURE, THEY WILL BE ENCOURAGED TO TAKE THE INFORMED CONSENT LETTER TO THEIR NEUROLOGIST OR FAMILY PHYSICIAN FOR FURTHER DISCUSSION PRIOR TO SIGNING THE FORM. ADDITIONALLY, AS PART OF THE TESTING SESSION, THE SCORES RELATED TO SEVERITY OF THE PARITICPANT WITH PARKINSON'S DISEASE WILL BE EVALUATED USING A SERIES OF DIRECT MOTOR EXAMINATIONS AND SELF-REPORT QUESTIONS, AND THIS INFORMATION CAN BE MADE AVAILABLE TO THE PHYSICIAN UPON THEIR REQUEST.Exercise Intervention:•The exercise intervention includes large magnitude movements, most difficult being forward and side-to-side lunges. These large magnitude movements may result in overuse injuries, such as sprains or strains, or muscle soreness. Additionally, since the duration of exercise sessions is approximately 1-hour, participants may become fatigued, especially individuals who are more sedentary. However, due to the low intensity nature of the program, and the slow velocity movements that will be promoted, these risks are minimal. Since the dual tasking PD-SAFEx group will be performing secondary cognitive and manual tasks while exercising, these individuals with Parkinson's disease will be at a greater risk of falling. To minimize this risk, participants will be encouraged to perform movements slowly, use the closest wall or chair for balance, and will be monitored by the lead instructor and

		volunteers. Participants will have the opportunity to rest at any time during the exercise program. The intensity of the program does not increase. However, the difficulty of exercise tasks and dual tasks will progressively increase in difficulty with subsequent weeks. This slow progression will familiarize participants with the exercise movements and acclimatize individuals to activity that may not be part of their daily lives prior inclusion in the exercise program. These aspects of the program with minimize the risk of injury.
6.3	Are there any potential social risks regarding this research (e.g., loss of privacy, loss of status, loss of reputation)? (If 'No' go to question 6.5)	Yes
6.4	If you marked 'Yes' to the above question provide your response here:	Participants will be recruited through a private PD database, in which their status as an individual with PD has been previously established.
6.5	Are there any potential psychological or emotional risks regarding this research (e.g., loss of self confidence after poor performance on a memory test, regret over the revelation of personal information to an interviewer, disruption of family routine, long waits, boredom, revelation of personal information)? (If 'No' go to question 6.7)	Yes
6.6	If you marked 'Yes' to the above question provide your response here:	Individuals may lose self-confidence if they are unable to complete the experimental tasks properly, if their performance is poor on the cognitive assessments, or if their progression through the exercise intervention is slower than anticipated.
6.7	If participants in this study are members of the organization being studied (e.g., employees of the company, members of a club, etc.), are there any repercussions by participating or not participating in this research? (If 'No' go to question 6.9)	No
6.8	If you marked 'Yes' to the above question provide your response here:	

6.9	Do participants in this research face risks other than those they would encounter in their everyday lives? (If 'No' go to the next question.)	Yes

6.10	If you marked 'Yes' to the above question provide your response here:	•There are no known risks of delaying medication (typically no longer than 14 hours), since it would be similar to how participants with Parkinson’s disease sleep through the night without taking any anti-Parkinson’s medication. Delaying medication might also feel similar to how individuals wit Parkinson’s disease might feel if they were to have forgotten to take a dosage of their medication during the day. In some cases, participants may not notice any differences at all. Although this medication delaying procedure does not need to be approved by a physician prior to the experiment, if the participants have any questions whatsoever regarding the risks of this delaying procedure, they will be encouraged to take the informed consent letter to their neurologist or family physician for further discussion prior to signing the form. Additionally, as part of the testing session, the scores related to severity of the participant with Parkinson’s disease will be evaluated using a series of direct motor examinations and self-report questions, and this information can be made available to the physician upon their request. •Some of the balance tasks that the participants are completing are not applicable to participant’s everyday lives (such as standing on the foam platform with the translucent goggles). This results in an increased risk of falling compared to participants every day lives. This risk will be minimized by the presence of a spotter to ensure the participant does not fall, is comfortable, and to help aid the participant in regaining postural stability in the event that they lose control of their balance. Half of the walking tasks in the pre-, post-, and washout will be completed while performing a secondary counting task, which may not be typical of the

		participants’ daily lives. To minimize the risk associated with this task, a spotter will walk to the side and slightly behind the participants throughout each walking trial, ready to lend help in the event that the participants lose control of their balance.<br>•The exercise intervention includes large magnitude movements, most difficult being forward and side-to-side lunges. These large magnitude movements may result in overuse injuries, such as sprains or strains, or muscle soreness, especially in individuals who may not be acclimatized to multiple bouts of physical activity per week. Additionally, since the duration of exercise sessions is approximately 1-hour, participants may become fatigued, especially individuals who are more sedentary. However, due to the low intensity nature of the program, and the slow velocity movements that will be promoted, these risks are minimal. Since the dual tasking PD-SAFEx group will be performing secondary cognitive and manual tasks while exercising, these individuals with Parkinson’s disease will be at a greater risk of falling. To minimize this risk, participants will be encouraged to perform movements slowly, use the closest wall or chair for balance, and will be monitored by the lead instructor and volunteers. Participants will have the opportunity to rest at any time during the exercise program. The intensity of the program does not increase. However, the difficulty of exercise tasks and dual tasks will progressively increase in difficulty with subsequent weeks. This slow progression will familiarize participants with the exercise movements and acclimatize individuals to activity that may not be part of their daily lives prior inclusion in the exercise program. These aspects of the

		program with minimize the risk of injury. In addition, since exercise may pose greater risk to individuals whose health status does not allow safe participation in physical activity (such as individuals with cardiovascular abnormalities), all participants recruited to participate in the study will be required to submit a Physical Activity Readiness Medical Examination (ParMed X), signed by a physician prior to joining the rehabilitation program.

6.11	If you marked 'Yes' to any of the questions above please explain what your plans are to minimize the risks you identified.	•Risk of losing postural control during balance tasks will be minimized by the presence of a spotter to ensure the participant does not fall, is comfortable, and to help aid the participant in regaining postural stability in the event that they lose control of their balance. To minimize the risk associated with walking tasks, a spotter will walk to the side and slightly behind the participants throughout each walking trial, ready to lend help in the event that the participants lose control of their balance. •The physical risk of the exercise will be minimized by promoting low intensity and slow velocity movements. Since the dual tasking PD-SAFEx group will be performing secondary cognitive and manual tasks while exercising, these individuals with Parkinson’s disease will be at a greater risk of falling. To minimize this risk, participants will be encouraged to perform movements slowly, use the closest wall or chair for balance, and will be monitored by the lead instructor and volunteers. Participants will have the opportunity to rest at any time during the exercise program. The intensity of the program does not increase. However, the difficulty of exercise tasks and dual tasks will progressively increase in difficulty with subsequent weeks. This slow progression will familiarize participants with the exercise movements and acclimatize individuals to activity that may not be part of their daily lives prior inclusion in the exercise program. These aspects of the program with minimize the risk of injury. In addition, since exercise may pose greater risk to individuals whose health status does not allow safe participation in physical activity (such as individuals with cardiovascular abnormalities), all participants recruited to participate in the

		study will be required to submit a Physical Activity Readiness Medical Examination (ParMed X), signed by a physician prior to joining the rehabilitation program.

7. Benefits to Participants

#	Question	Answer

7.1	What are the likely benefits of the research to the researcher(s), the participants, the research community and society at large that would justify asking people to participate in your project?	Since the exercise intervention that is being utilized has previously demonstrated significant improvements to symptom severity and the progression of Parkinson's disease, participants in the exercise groups may receive direct benefits to the movement symptoms associated with Parkinson's disease. Additionally, with improvements in symptoms, this might positively influence activities of daily living and the quality of life in those who participate. Although evidence has not be collected to support that PD-SAFEx improve cardiovascular capacity or muscular strength, due to the nature of the exercise, and the sedentary lifestyle that participants may have been used to prior to participating in the present exercise study, cardiovascular and muscular strength benefits may be provided to participants. Additionally, since the exercise program is conducted in a group setting, participants will have the opportunity to socialize with other individuals that share the experience of living with Parkinson's disease, also providing potential benefits to quality of life. In addition to providing improvements to symptoms of Parkinson's disease, this study will provide a greater understanding into the pathophysiological mechanisms underlying improvements associated with exercise rehabilitations employed for Parkinson's disease. To date, goal-directed exercises have yet to investigate the rudimentary aspects of the exercise, since no studies have directly compared identical exercise regimens while focusing on task irrelevant information as opposed to task relevant information. Furthering understanding into this realm of research will help find the most effective exercise intervention to improve symptoms in Parkinson's disease, slow disease

		progression, and improve quality of life.
7.2	Explain why these benefits outweigh any risks you may have identified in Section 6.	Participation in this study will improve symptom severity in those individuals who are randomized to the two exercise groups. These improvements have previously been shown to positively correlate with improved quality of life, and therefore risk of falling and injury is argued to be outweighed by the direct benefits participants will receive. Furthermore, with improvements in postural control and balance are often associated with improvements in symptom severity, so as the intervention progresses from week to week, the risk of falling and injury will decline and also benefit the individuals' daily lives. All participants will also aid in substantially improving understanding of the efficacy of exercise as rehabilitation for symptomatic alleviation in Parkinson's disease, which will lend more understanding to the progression of Parkinson's disease, which is not completely understood.

8. Deception and Concealment

#	Question	Answer
8.1	Is any deception (the act of deliberately misleading participants) or concealment (the act of keeping information from participants without deceiving them) necessitated by the study's design? (If 'No' go to the next tab above "Privacy and Confidentiality.")	No
8.2	If 'Yes' to the above question, describe and justify below the deception or concealment being used. Attach a copy of the debriefing statement to be used immediately afterward using the 'Attachments' Tab above.	

9. Privacy and Confidentiality

#	Question	Answer

9.1	If it is necessary to protect the identity of participants during the conduct of the research, how will this be done?	All participant data and results will be coded, using a coding system. Data will be stored on a secure computer protected by password with data coded in encrypted files, and all paper documents (including consent forms) will be kept in an alarm secured room within the Sun Life Financial Movement Disorders Research and Rehabilitation Centre.
9.2	If applicable, how will individual participants remain anonymous and unidentifiable in the publication and other release of study findings?	Results will be released and published as group effects. If the need arises in which an outlier must be acknowledged, the participants will only be identified by their identification number. There will be no association made between the participant's assigned identification number and any personal data/information that could identify the individual.
9.3	Describe how you will ensure confidentiality of all data or information collected from participants.	Each individual participant will be assigned an identification number. Only the student investigator, Eric Beck, will be aware of the association between the coding system, participant's identification number, personal information given, and the experimental data collected. Experimental data will be stored separately from the personal information of the participants.
9.4	Who will have access to the data collected from participants?	The primary investigator, Eric Beck, and supervisor Dr. Almeida will have access to the data collected from participants.

9.5	How long will the data be retained? Describe how the data will be disposed of and who will be responsible for the disposal of the data.	Personal information and experimental data will be retained indefinitely 4. FOR THREE REASONS: 1) FOR THE ABILITY TO RETRO-ACTIVELY ANALYZE PARTICIPANT DATA WITH REGARDS TO KNEW LONGITUDINAL RESEARCH QUESTIONS, 2) TO TRACK THE PROGRESSION OF DISEASE SEVERITY IN PARTICIPANTS THAT CONTINUE TO PARTICIPATE IN STUDIES (THIS WILL ALLOW US TO SCALE EXERPERIMENTS TO THE PARTICIPANTS' ABILITIES AND NEEDS AS WELL AS PROVIDE THE PARTICIPANT WITH INFORMATION REGARDING THE DISEASE WHICH THEY MAY WISH TO DISCUSS WITH THEIR HEALTH-CARE PROVIDER), AND 3) PARTICIPANT CONTACT INFORMATION IS PART OF THE MDRC PARTICIPANT DATABASE AND USED TO CONTACT PARTICIPANTS IN THE FUTURE UNTIL PARTICIPANTS NO LONGER WISH TO BE IN THE DATABASE.
9.6	Will quotes from participants be used in any write-ups or presentations? If so, participants must be told in the information/informed consent statement that quotations may be used. They must also be told whether or not any quotations could allow them to be identified. Participants should also be given the chance to vet their quotations before being used. Will participants be quoted? (If 'No' proceed to next tab 'Compensation of Participants)	No
9.7	If you marked 'Yes' to the above question, will participants be identifiable in these quotations? If not, how will you ensure this?	

9.8	Can participants consent to taking part in the project as a whole but not having their quotations used in the final report? If so, add a separate line to the end of the consent statement relating directly to the use of quotations.	

10. Compensation of Participants

#	Question	Answer
10.1	Will participants be rewarded or compensated, financially or otherwise? (If 'No' go to the 'Conflict of Interest' tab)	No
10.2	If you marked 'Yes" to the above question please provide details of and justification for the compensation being offered.	

11. Conflict of Interest

#	Question	Answer
11.1	Describe any conflict(s) of interest (actual, perceived, or potential) that you or anyone else associated with this project have relating to this project. Refer to Laurier’s conflict of interest policy (go to the 'Useful Links' tab on your home screen; select 'Laurier - Conflict of Interest Policy.') If there is a conflict of interest, describe how and when you will disclose it to your participants during the free and informed consent process. ENTER BELOW “N/A” IF THERE IS NO CONFLICT.	N/A

12. Ethical Training of the Researcher(s)

#	Question	Answer
12.1	Researchers are responsible for ensuring that all individuals associated with this project know and comply with all the University's guidelines for ethical research. Outline below the measures planned (or already taken) to conduct or confirm the ethical training of all such personnel involved with this project.	All personnel have completed the Tri-Council Policy Statement: Ethical Conduct For Research Involving Humans Course on Research Ethics (TCPS 2: Core).

12.2	I/we have read the University's current guidelines for the ethical conduct of research involving human participants, available on the Office of Research Services webpage and agree to comply with them.	Yes

13. Feedback to Participants

#	Question	Answer
13.1	Will participants be debriefed after their participation? (If 'Yes' attach a copy of the debriefing statement under the 'Attachments' Tab.)	No
13.2	Will feedback regarding the study's findings be provided to the participants? If 'Yes," please provide the date by which the results will be available.	No
13.3	If participants will receive feedback, explain how the participants will receive the information.	Participants will receive an updated summary of the findings from this study by way of an annual handout or luncheon event from the MDRC that will update the participants on all of the research being conducted at the MDRC. Additionally, participants are welcomed to visit the MDRC and view hung posters that summarize findings of each study conducted at the MDRC.
13.4	Do you plan to publish and/or present the findings of this study, and/or provide the results to any other agency or organization?	Yes
13.5	If you marked 'Yes' to the above question, describe the place(s) where the results of the study may be published and/or presented (e.g., conference, presentation, workshop, book, journal, website, thesis, etc.), as well as list the organizations and agencies that will receive a copy of the results.	The results of this study may be published in journal articles and presented at conferences.

14. Supervisor Sign Off (Required only for Students)

#	Question	Answer

14.1	By checking "Yes" below I am confirming that I have attached my supervisors sign off on this REB application and that I have attained all necessary department or faculty approvals for this research.	Yes – Supervisors email sign off has been attached

15. TCPS2 Tutorial Certificate Requirement

#	Question	Answer
15.1	All researchers listed within an REB application (e.g., PI, co-PI, supervisor, students, research assistants) must complete the online TCPS2 tutorial. A copy of the certificate of completion for each researcher must be uploaded under the "Attachments" Tab prior to submission to the REB. A link to the tutorial is provided under the "Attachments" Tab.	Yes - As PI I have attached all required TCPS tutorial certificates

Attachments

Doc / Agreement	Version Date	File Name	Description
Annual or Final Report	2016/08/31	Annual Report.pdf	Annual Report Supervisor Sign off
Consent / Information Letter	2015/06/16	MSc Informed Consent_EricBeck.docx	Informed Consent
Consent / Information Letter	2015/06/22	MSc Informed Consent_EricBeck_Revisions.docx	Revised Informed Consent
Consent / Information Letter	2015/08/05	ee21508e-3ac1-4a80-9fb9-36107d9b0608_MSc Informed Consent_EricBeck_Revisions.docx	Modified Consent Form
Other	2015/06/16	Dual tasks to be employed.docx	Dual Tasks to be Employed
Other	2015/06/16	PD-SAFEx Weekly Exercises.docx	PD-SAFEx Weekly Exercises

Other	2015/06/16	MSc Recruitment of Participants Phone Script_Eric Beck.docx	Recruitment Phone Script
Other	2015/08/05	bda00ece-810a-4680-a2a6-58996f02aa85_MSc Recruitment of Participants Phone Script_Eric Beck.docx	Modified Phone Script
Questionnaire/Survey	2015/08/05	Physical Activity Group Environment Questionnaire.docx	Physical Activity Group Environment Questionnaire
Questionnaire/Survey	2015/08/05	BREQ-3.docx	Exercise Regulations Questionnaire
Questionnaire/Survey	2015/08/05	State Trait Anxiety Inventory for adults.pdf	State-Trait Anxiety Inventory For Adults
Questionnaire/Survey	2015/08/05	Parkinson Anxiety Scale.doc	Parkinson's disease Anxiety Scale
Questionnaire/Survey	2015/08/05	The Movement Reinvestment Scale.pdf	The Movement Specific Reinvestment Scale
Supervisor's sign off	2015/06/15	Supervisor Sign Off Email.pdf	Supervisor Sign Off Email
Supervisor's sign off	2015/08/05	Supervisor Modification Sign Off Email.pdf	Supervisor Sign Off
TCPS2 Tutorial Certificate	2013/08/09	TCPS 2 Certificate-Eric Beck.pdf	Eric Beck TCPS Certificate
TCPS2 Tutorial Certificate (Supervisor's)	2013/05/06	tcps2_core_certificate_QUINCY.pdf	Supervisor TCPS Certificate
