# Supplementary material for: Sensory focused exercise improves anxiety in Parkinson’s disease: A randomized controlled trial
Source: PLoS One. 2020 Apr 16;15(4):e0230803. doi: 10.1371/journal.pone.0230803 (PMC7162490; doi:10.1371/journal.pone.0230803)
Supplement: S2 Data — (DOCX) [file pone.0230803.s004.docx]

**MODIFICATIONS:**

- 1. **Changes to procedures or methods:**

With respect to the REB# 4542 accepted methodology, this modification proposes to add one experimental task, one additional measure of executive function, and five questionnaires to the procedures of the pre, post, and washout assessments.

**Added Task: Limb Matching Task to Measure Proprioception.** The added task will be completed by individuals with Parkinson’s disease in all three groups (ie. The original PD-SAFEs group, the dual tasking PD-SAFEx group, and the control group). Using a protocol similar to Zia (2000), the experimenter will guide the participants “reference arm” to a desired position (i.e. 50° or 90° verified by a goniometer), where the participants will hold the position by actively contracting their muscles. While their reference arm is held at the desired angle, participants will be instructed to actively flex their “matching arm” into the same angular position as the “reference arm.” Participants will be asked to hold the position for 3 seconds. Participants will be blind-folded for the full duration of this task and complete 6 randomized matching trials (3 trials for each angle) with the left and the right limb in a counterbalanced order (half of the participants starting with the right arm and the other half starting with the left arm). Thus, there will be 12 trials in total. Participants will be provided the opportunity to practice the task prior to the commencement of the experimental trials to ensure that instructions and protocol are fully understood. Optotrak® Certus™ Motion Capture System will be utilized to allow the capture or precise limb position coordinates, using two cameras. Participants will be seated in a chair with their forearms resting in a supinated position on a table. Four Optotrak^®^ infrared light emitting diodes will be attached to the lateral epicondyle of the humerus and the lateral aspect of the styloid process of the radius on both arms. The use of landmarks will ensure consistent placement of the markers. This task will add 15 minutes onto the data collection duration at the pre, post, and washout assessments.

**Added Measure of Executive Function: Verbal Fluency Task.** In this task, the experimenter will provide the participant with a letter (for example the letter S), and the participant will be asked to name as many words as they are capable of in 60 seconds that begin with the letter that the experimenter provided. The number of words will be recorded with a pencil on a data collection sheet and transcribed to an electronic data collection form for further analysis.

**Added Self-Assessment Questionnaires at the end of the accepted Tasks 3 and 5 from the REB# 4525 methodology:** At the end of each balance trial and walking trial in tasks 3 and 5, respectively, two Likert-type scales will be presented to the participant. With respect to the first scale, a 9-point *Self Assessment Manikin* scale will be presented to the participant, and the participant will be asked to inform the investigator their level of anxiety while balancing or walking in the previous trial, as measured from 1 (meaning no anxiety felt while completing the task) to 9 (the greatest level of anxiety the individual could imagine). With regards to the second scale, the participant will be asked to self-assess their level of mental engagement towards the task as either: *1=completely on the task, 2= mostly on the task, 3=both on the task and thinking about unrelated concerns, 4=mostly thinking about unrelated concerns, or 5=completely thinking about unrelated concerns.* These self-assessment scales will provide a measure of anxiousness while completing the tasks as well as their mental engagement.

**Added Tools For Data Collection: Audio Recording.** With respect to the already accepted *Stroop Test* measure of executive function and the proposed *Verbal Fluency Task,* since participants will be verbally announcing answers throughout the task at variable rates, and part of the scoring process of these tasks will be to measure accuracy of answers, the present modification proposes to use an audio recording device to record answers given by the participants. This will aid in proper scoring and analysis of the collected data. The audio device will only be turned on and commence recording when the participant begins the task, and therefore no Private/Personal information (ie. Identification of the participant) will be recorded. To ensure that participants’ agree to the use of the audio recording device, the following sentence has been added to the modified informed consent found in the attachments section: “I am aware that an audio recording will be capturing my voice, and it will be used for research purposes. With this knowledge I consent to audio recording”. On the informed consent, this will be followed by a space for a signature and date to be signed by the participant.

**Added Questionnaires:** Participants will be asked to additionally complete the pencil-and-paper questionnaires discussed below in order to answer two additional questions that have yet to be investigated in literature; whether a group setting exercise class intervention has the ability to influence specific exercise psychology motivations in individuals with Parkinson’s disease, and whether the present intervention can accomplish aspects that improve adherence to exercise programs in these individuals. These questionnaires will further be utilized to evaluate whether training attention with exercise in Parkinson’s disease might modify anxiety, a very prominent non-movement symptom found in Parkinson’s disease. Participants will be asked to complete these questionnaires at pre, post, and washout assessment periods of the present proposed exercise intervention. The added time duration to complete these questionnaires will be an additional 30 minutes. Individuals from each of the three groups will be asked to complete the questionnaires. The added questionnaires can be found in the attachments section.

**Questionnaire 1: Physical Activity Group Environment Questionnaire.** This questionnaire is a scaled measurement of the propensity in which participants are attracted to the exercise intervention for specific reasons, whether they are attracted to the individual exercise tasks, the social aspect of the exercise, or the requirement to complete the tasks as a group. This questionnaire will provide a measurement to investigate the aspects of the exercise program that attracts individuals with Parkinson’s disease to adhere to the intervention. With this information, we aim to provide recommendations with respect to the aspects of exercise that should be involved in prescribed exercise programs to optimize adherence for individuals with Parkinson’s disease.

**Questionnaire 2: Exercise Regulations Questionnaire.** This questionnaire will be utilized in addition to the *Physical Activity Group Environment Questionnaire* to further investigate the aspects of an exercise program that attract individuals with Parkinson’s disease to adhere to the intervention.

**Questionnaire 3: State-Trait Anxiety Inventory for Adults.** The purpose of this validated questionnaire is to measure the degree in which participants are anxious at the time of data collection (State), as well as the degree to which they feel anxious in general (Trait). This will provide indications as to whether training attention (by either having participants with Parkinson’s disease focus on sensory feedback or on a dual task) can be utilized to improve anxiety in individuals with Parkinson’s disease.

**Questionnaire 4: The Parkinson’s Anxiety Scale.** This questionnaire will be utilized in addition to the *State-Trait Anxiety Inventory for Adults* to further investigate whether training attention in addition to the exercise has the propensity to modify anxiety in Parkinson’s disease. Together, these scaled questionnaires will allow us to make recommendations as to whether these forms of exercises could be prescribed to improve anxiety in Parkinson’s disease.

**Questionnaire 5: The Movement Specific Reinvestment Scale.** Movement Reinvestment refers to phenomenon in which individuals with a greater likelihood to fall, such as individuals with Parkinson’s disease, require greater conscious control of their movements to ensure accuracy of movement. Although this compensatory behaviour is beneficial to decrease the chance of falling, individuals who employ reinvestment lose the ability to complete multiple processes simultaneously, such as walking while talking. This phenomenon has been linked to anxiety, and therefore in addition to the previous anxiety questionnaires, *The State-Trait Anxiety Inventory for Adults and the Parkinson’s Anxiety Scale,* this questionnaire will provide an knowledge as to whether the proposed intervention might effect anxiety, negating participant’s need to reinvest.

**Risks associated with the additional tasks:** There are no further risks associated with the additional tasks, executive measures, or the added questionnaires that were not previously discussed in the protocol that has been accepted (REB#4455). Participants may become fatigued due to the number of questionnaires. There is no additional physical risk associated with the limb-matching task.

**Minimizing the risks:** In the event that individuals feel fatigued as a result of the number of questionnaires they will be asked to complete, participants will be encouraged to inform the primary investigator, Eric Beck, and take as many breaks as they require, for a duration they feel is suitable.

### 1.5 Add a new (or revised) consent letter (Explain why this change is needed - use the "Attachment" tab to attach the consent letter; underline the changes you made in the consent letter.)

### The change is needed to inform the recruited participant of the additional number of tasks and trials that will be completed, the description of the added tasks, as well as inform the participant of the added questionnaires to be completed.

### 1.6 Add a new (or revised) oral script (Explain why this change is needed - Use the "Attachment" tab to attach the oral script; underline the changes you made in this document.)

### The change is needed to inform the prospective recruited participant of the additional number of tasks and trials that will be completed, as well as the description of the added tasks.
